# Engineering strategies for enhanced 1′, 4′-*trans*-ABA diol production by *Botrytis cinerea*

**DOI:** 10.1186/s12934-024-02460-8

**Published:** 2024-06-26

**Authors:** Yifan Wang, Dan Shu, Zhemin Li, Di Luo, Jie Yang, Dongbo Chen, Tianfu Li, Xiaonan Hou, Qi Yang, Hong Tan

**Affiliations:** 1 CAS Key Laboratory of Environmental and Applied Microbiology, Environmental Microbiology Key Laboratory of Sichuan Province, Chengdu Institute of Biology, Chinese Academy of Sciences, Chengdu, China; 2 University of Chinese Academy of Sciences, Beijing, China

**Keywords:** *Botrytis cinerea*, Metabolic engineering, 1′,4′*-trans-*ABA-diol, Overexpression

## Abstract

**Background:**

Currently, industrial fermentation of *Botrytis cinerea* is a significant source of abscisic acid (ABA). The crucial role of ABA in plants and its wide range of applications in agricultural production have resulted in the constant discovery of new derivatives and analogues. While modifying the ABA synthesis pathway of existing strains to produce ABA derivatives is a viable option, it is hindered by the limited synthesis capacity of these strains, which hinders further development and application.

**Results:**

In this study, we knocked out the *bcaba4* gene of *B. cinerea* TB-31 to obtain the 1′,4′-*trans*-ABA-diol producing strain ZX2. We then studied the fermentation broth of the batch-fed fermentation of the ZX2 strain using metabolomic analysis. The results showed significant accumulation of 3-hydroxy-3-methylglutaric acid, mevalonic acid, and mevalonolactone during the fermentation process, indicating potential rate-limiting steps in the 1′,4′-*trans*-ABA-diol synthesis pathway. This may be hindering the flow of the synthetic pathway. Additionally, analysis of the transcript levels of terpene synthesis pathway genes in this strain revealed a correlation between the *bchmgr*, *bcerg12*, and *bcaba1-3* genes and 1′,4′-*trans*-ABA-diol synthesis. To further increase the yield of 1′,4′-*trans*-ABA-diol, we constructed a pCBg418 plasmid suitable for the *Agrobacterium tumefaciens*-mediated transformation (ATMT) system and transformed it to obtain a single-gene overexpression strain. We found that overexpression of *bchmgr*, *bcerg12*, *bcaba1*, *bcaba2*, and *bcaba3* genes increased the yield of 1′,4′-*trans*-ABA-diol. The highest yielding ZX2 A3 strain was eventually screened, which produced a 1′,4′-*trans*-ABA-diol concentration of 7.96 mg/g DCW (54.4 mg/L) in 144 h of shake flask fermentation. This represents a 2.1-fold increase compared to the ZX2 strain.

**Conclusions:**

We utilized metabolic engineering techniques to alter the ABA-synthesizing strain *B. cinerea*, resulting in the creation of the mutant strain ZX2, which has the ability to produce 1′,4′-*trans*-ABA-diol. By overexpressing the crucial genes involved in the 1′,4′-*trans*-ABA-diol synthesis pathway in ZX2, we observed a substantial increase in the production of 1′,4′-*trans*-ABA-diol.

**Supplementary Information:**

The online version contains supplementary material available at 10.1186/s12934-024-02460-8.

## Background

*Botrytis cinerea* is common necrotrophic plant-pathogenic fungus that can infect over 1000 plant species worldwide [1]. Due to its significant impact on agricultural production. It has become a key model fungus for studying the necrotrophic pathogens [2]. The *Botrytis* genus is capable of producing various secondary metabolites, including abscisic acid (ABA), glucanases, polysaccharides, and the plant toxin botrydial [3–6]. ABA is a well-known phytohormone, a sesquiterpene that plays a crucial role in drought and salinity tolerance [7]. While ABA is commonly found in the metabolites of higher plants, lichens, bryophytes, and algae, it is not specific exclusive to plants and has also been identified in fungi such as the *Cercospora* and *Botrytis* genera [8, 9]. *B. cinerea* has demonstrated promising potential as a candidate for industrial production of ABA, thanks to its ability to produce high yields of the hormone in a short period of time. In our previous studies, we isolated wild-type *B. cinerea* strains from wheat stems and leaves in southwest China [10]. Since then, we have generated several mutants with varying ABA yields through compound mutagenesis. These mutants have been utilized in studies to gain a better understanding of the regulation of ABA biosynthesis and its potential for industrial production [11, 12].

**Fig. 1:**
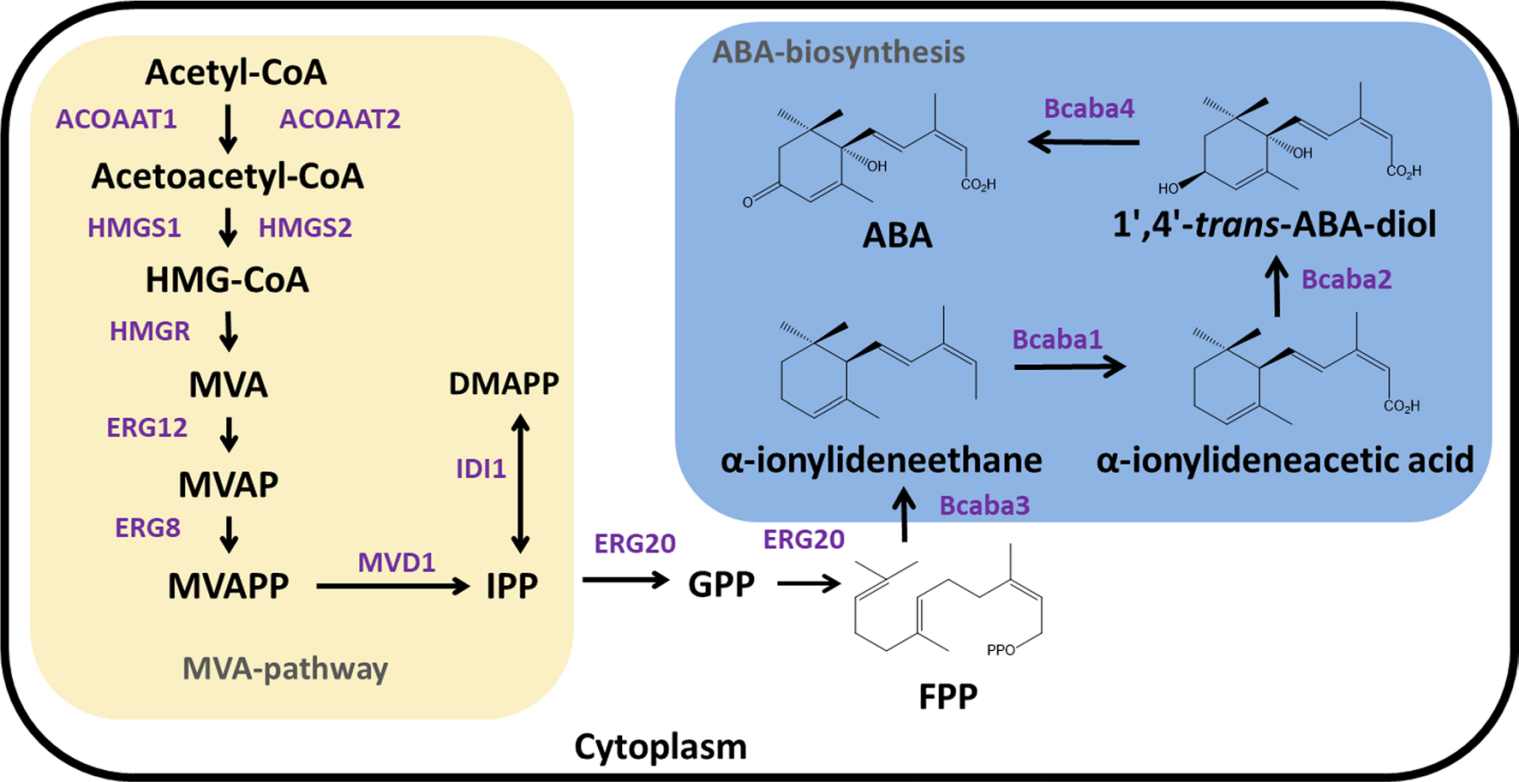
Synthesis pathway of ABA in *Botrytis cinerea*. The solid and dashed arrows represent single and multiple enzyme reactions. ACOAAT1:acetyl-CoA acetyltransferase 1; ACOAAT2: acetyl-CoA acetyltransferase 2; HMGS 1, 3-hydroxy-3-methylglutaryl-CoA synthase 1; HMGS2, 3-hydroxy-3-methylglutaryl-CoA synthase 2; HMGR: 3-hydroxy-3-methylglutaryl-CoA reductase; ERG12: mevalonate kinase; ERG8: phosphomevalonate kinase; MVD1: diphosphomevalonate decarboxylase; IDI1: isopentenyl-diphosphate delta-isomerase; ERG20: farnesyl diphosphate synthase. BcABA1: *B. cinerea* cytochrome P450; BcABA2: *B. cinerea* cytochrome P450; BcABA3: *B. cinerea* α-ionylideneethane synthases; BcABA4: *B. cinerea* dehydrogenase. HMG-CoA, 3-hydroxy-3-methylglutaryl-CoA; MVA, mevalonic acid; MVAP, phosphomevalonate; MVAPP, diphosphomevalonate; IPP, isopentenyl diphosphate; DMAPP, dimethylallyl diphosphate; GPP, geranyl diphosphate; FPP, farnesyl diphosphate

ABA belongs to the group of sesquiterpenoid, and its synthesis follows the same characteristics as other terpene. Terpenes are all composed of isoprene, and the central intermediate in their biosynthesis is isopentenyl diphosphate (IPP) [13]. There are two distinct pathways for IPP biosynthesis in nature; in most bacteria, algae, and plant plastids, IPP is synthesized through the deoxyxylulose-5-phosphate (DXP) pathway (also known as MEP pathway), using pyruvate and glyceraldehyde-3-phosphate as precursors [14]. In recent years, the ABA biosynthetic pathway in *B. cinerea* has been extensively studied (Fig. 1) [15]. Initially, isopentenyl diphosphate (IPP) is produced through multiple enzymatic reactions in the MVA pathway [16]. Then IPP is combined with dimethylallyl diphosphate (DMAPP) in a head-to-tail manner, catalyzed by the farnesyl pyrophosphate synthase, to form geranyl diphosphate (GPP). GPP is then sequentially combined with another IPP unit to ultimately produce the C_15_-sesquiterpenes precursor, farnesyl diphosphate (FPP) [17]. Finally, a cluster of ABA synthesis genes (*bcaba1*-4) is responsible for the later stages of ABA biosynthesis. The sesquiterpene synthases (Bcaba3) converts FPP, which is formed from the condensation of the three IPP molecules, into α-ionylideneethane [18]. It is then oxidized by *B. cinerea* cytochrome P450 (Bcaba1) and cytochrome P450 (Bcaba2) to form 1ʹ,4ʹ-*trans*-dihydroxy-α-ionylideneacetic acid (1ʹ,4 ʹ-*trans*-ABA-diol), which is finally reduced by dehydrogenase (Bcaba4) to form ABA [19, 20]. It has been discovered that the sesquiterpene cyclase (STC) encoded by the *bcstc5* gene is also involved in ABA synthesis, leading to the proposal of an ABA cluster consisting of five genes [21]. However, it has been demonstrated that the expression of *bcaba1-4* in heterologous hosts is sufficient for ABA synthesis [22]. The role of the *bcstc5*/*bcaba5* genes in ABA synthesis has yet to be confirmed.

1ʹ,4ʹ-*trans*-ABA-diol is a derivative of ABA with a hydroxyl group only at the 4' position. Its chemical synthesis was first documented in 1972, and later studies by Hirai et al. confirmed its presence in *B. cinerea* culture filtrates and its role as an endogenous precursor of ABA [23, 24]. Other fungi have also been found to use 1ʹ,4ʹ-*trans*-ABA-diol as a biosynthetic precursor of ABA [25, 26]. However, it has been found to not be a precursor of ABA in higher plants [27, 28]. Studies have demonstrated that 1ʹ,4ʹ-*trans*-ABA-diol inhibits the growth of excised embryonic bean axes growth and rice seed germination [23, 29]. When sprayed on tobacco seeding, it has been found to increase the expression of abiotic stress tolerance genes and potential biotic resistance genes in tobacco seedlings, resulting in physiological effects similar to those of exogenous ABA [30]. While it is possible that 1ʹ,4ʹ-*trans*-ABA-diol is converted to ABA in vivo to exert its effects, it is more likely that the substance itself is physiologically active. The 4'-methoxy derivative of 1ʹ,4ʹ-*trans*-ABA-diol, which has no diminished ABA-like activity, has been used to produce molecular probes for detecting ABA-binding proteins [31]. Previous studies have primarily focused on the physiological activities and catabolic processes of 1ʹ,4ʹ-*trans*-ABA-diol due to the challenges in obtaining high purity and large quantities. However, there have been no reported studies on its fermentation. Given its structure and biological activity, there is a significant need for further exploration of its potential and value.

This paper reports on the engineering and optimisation of *B. cinerea* for the production of 1ʹ,4ʹ-*trans*-ABA-diol. By genetic engineering, we obtained the strain ZX2, which is capable of producing 1ʹ,4ʹ-*trans*-ABA-diol.Based on the analysis of the metabolomics of the fermentation broth and the transcript expression levels of the genes involved in the synthetic pathway, gene overexpression was conducted, resulting in the generation of a strain with significantly enhanced capability for the synthesis of 1ʹ,4ʹ-*trans*-ABA-diol.This work will provide valuable insights for developing strategies to utilize *B.cinerea* as a microbial cell factory for sustainable and economical production of valuable analogues of ABA.

## Materials and methods

### Strains, plasmids and primers

*B.cinerea* TB-31 strain was laboratory preserved [32]. *Escherichia coli* DH5α was used for plasmid construction and *Agrobacterium tumefaciens* EHA105 was used for transform plasmid DNA. Plasmids and strains used in this study are listed in Tables 1 and 2, respectively. All primers pairs used in this study were designed using the Primer Premier 5 (Premier Biosoft International, Palo Alto, CA, USA) software, and were synthesized by Tsingke (Table S1, S2).

**Table 1:** List of plasmids

Plasmid	Description	Source
pCBh1	*B. cinerea*-integrative plasmid, PAnOliC-TAntrpC, hph	Ding et al., [33]
pSilient-Dual1	Filamentous fungal RNA silencing plasmid, neo	MiaoLing Biology
pCBg418	*B. cinerea*-integrative plasmid, PAnOliC-TAntrpC,G418	This study
pCBHR	PAnOliC-HMGR-TAntrpC, G418	This study
pCBMK	PAnOliC-ERG12-TAntrpC, G418	This study
pCBFS	PAnOliC-ERG20-TAntrpC, G418	This study
pCBA1	PAnOliC-ABA1-TAntrpC, G418	This study
pCBA2	PAnOliC-ABA2-TAntrpC, G418	This study
pCBA3	PAnOliC- ABA3-TAntrpC, G418	This study

**Table 2:** List of stains

Strains	Description	Source
*B. cinerea* TB-31	ABA synthesis strains	Our lab
*B. cinerea* ZX2	Δ*bcaba4*	This study
ZX2 HR	Δ*bcaba4*,overexpressing *bchmgr*	This study
ZX2 MK	Δ*bcaba4*, overexpressing *bcerg12*	This study
ZX2 FS	Δ*bcaba4*, overexpressing* bcerg20*	This study
ZX2 A1	Δ*bcaba4*, overexpressing* bcaba1*	This study
ZX2 A2	Δ*bcaba4*, overexpressing* bcaba2*	This study
ZX2 A3	Δ*bcaba4*, overexpressing *bcaba3*	This study

### Media and fermentation conditions

Bacteria were incubated in LB plates containing 50 μg/mL kanamycin at 37 °C.The TB-31 strain was cultured on potato dextrose agar (PDA) medium, ZX2 strain was cultured on PDA medium containing 50 μg/mL of hygromycin B, and the transformed strains were cultured on PDA medium containing hygromycin B and 160 μg/mL of G418 sulphate. Seed cultivation of *B.cinerea* strains were carried out in seed medium (50 g/L wheat bran extract, 10 g/L glucose, 0.5 g/L potassium dihydrogen phosphate, 3 g/L sodium carboxymethylcellulose, 0.1 mg/mL vitamin B1 1 mL, pH natural).Fermentation medium containing 40 g/L wheat bran extract, 10 g/L glucose, 0.5 g/L potassium dihydrogen phosphate, 0.1 mg/mL vitamin B_1_ 1 mL.

The *B.* *cinerea* strains were activated for 8 days in PDA medium at 25 °C. For the preparation of seed culture, 1.5 mL spore suspension (10^7^ conidia/mL) was inoculated into 250 mL flasks containing 40 mL seed medium and culture at 25 ℃, 200 rpm for 48 h. Following that, 5 mL of seed culture was added to a 250 mL flask containing 50 mL of fermentation medium and cultured at 25 ℃, 200 rpm for 48 h. As for fed-batch fermentation, 1.5 L of seed culture was inoculated into 50 L fermenter containing 25 L fermentation medium. The sucrose feed (80 g/L) was fed according to the rate of reducing sugar consumption from 42 to 162 h. The incubation temperature was 25 °C, agitation speed 200–400 rpm, tank pressure 0.045–0.048 MPa, concentration of dissolved oxygen ranged 10–70%, pH 6–8, and fermentation for 7 days.

### Construction ZX2 strain and mutants strains

We generated a knockout mutant of *bcaba4*. The *bcaba4* gene knockout vector was constructed using the double-joint PCR method [34]. The Genomic DNA was extracted with the E.Z.N.A. Fungal DNA Mini kit (Omega, D3390-01, USA). With the DNA of TB-31 as the template, we amplified the 5′ fragment (620 bp) and 3′ fragment (524 bp) of the open reading frame (ORF) of *bcaba4* by the primer pairs *bcaba4*-5′F/-R and *bcaba4*-3′F/-R, respectively. The* hyg* fragment was amplified using primer pairs *hyg*-F/-R. With the primer pair *bcaba4*-5′F/3′-R to amplify the gene knockout cassette by overlapping PCR. *B. cinerea* TB-31 strain was cultured on PDA medium at 25 °C for 8–9 days and collected spores were digested in 1% Lysing Enzymes from *Trichoderma Harzianum* (Sigma-Aldrich) in KC solution at 25 °C, 60 rpm shaking for 1 h, adjusted to 1 × 10^6^ conidia·mL^−1^. The protoplast method transforms knockout cassettes [35]. Transformants were selected on PDA containing hygromycin B(50 μg/mL), and were verified by PCR using the primers P7/P8, P9/P10, P5/P6, P7/P11(Table S1)and driven by real-time quantitative reverse transcription PCR (qRT-PCR) using the primers *bcaba4*-F/-R.

We amplified overexpressed genes cDNA from *B. cinerea* ZX2 using the primers target-F/-R, and cloned into the *Xba*I and *Sca*I digested sites in pCBg418 to generate six vector, respectively [36]. Overexpression strains were constructed using ATMT, transformants were selected on PDA that contained hygromycin B(50 μg/mL) and G418 sulfate (160 μg/mL), and three generations were purified on PDA with hygromycin and G418 sulfate [36]. The transformants were verified by PCR using the primers test-F/test-R, *neo*-F/-R, and qRT-PCR using the primers qRT-target-F/-R. All transformants were purified for three generations on the corresponding resistant PDA.

### Sensitivity determination and plasmid construction

Bleomycin (Macklin), G418 (Aladdin) and Nourseothricin (Shanghai Maokang) preparations were prepared in sterile water, and a solution of Benomyl (Macklin) preparation was prepared in acetone, diluted to the appropriate concentration and added to PDA medium at 50–55 °C, mixed and poured onto plates (Table.S3). The ZX2 strain was inoculated onto PDA dishes containing different concentrations of antibiotics and incubated at 25 °C for 5 days to observe the growth of the strain. The incubation was repeated 3 times on each antibiotic concentration.

The pSilient-Dual1 plasmid was used as a template to amplify the neo resistance gene (using Phanta Max Super-Fidelity DNA Polymerase, Vazyme), along with the AnTrpC promoter (promoter) and CaMV35S polyA signal (terminator) fragments of the pCBh1 plasmid. And sequentially ligated to the upstream and downstream of the resistance gene to construct resistance expression cassettes. The pCBh1 was linearised with *Pme*I and *Xmn*I (using Restriction Endonuclease, New England Biolabs) and recombined with the *neo* expression cassette (using ClonExpress MultiS One Step Cloning Kit, Vazyme) to construct the pCBg418 vector. The vector is G418 resistant and operates at the polyclonal site MCS. All gene were annotated with the NCBI genome database of *B.cinerea* strain B05.10 used in primer design. The pCBg418 was first linearized with *Sac*I/*Xba*I and then was recombined with the genes. The genes HMGR, MK, ABA1, ABA2 and ABA3 were cloned into pCBg418 (Fig. S5) with primers HMGR-F/HMGR-R, MK-F/MK-R, ABA1-F/ABA1-R, ABA2-F/ABA2-R and ABA3-F/ABA1-R to yield plasmid pCBHR, pCBMK, pCBA1, pCBA2 and pCBA3, respectively. All constructed plasmids were transferred into DH5α separately and the correct transformants were identified by sequencing.

### Metabolomic analysis by UHPLC-MS

The fermentation broth was centrifuged at 10,000*g*, 4 °C for 3 min and 1 mL of supernatant is freeze-dried. Then the dried residue was dissolved in 100 μL of methanol/water (1:4, v/v) and incubated on ice for 5 min before centrifugation at 15,000*g*, 4 °C for 15 min. Finally, a portion of the supernatant was diluted with LC–MS grade water to 53% methanol and centrifuged at 15,000*g*, 4 °C for 15 min. The supernatant was collected and injected into UHPLC-MS for analysis [37]. The quantitative detection of metabolomics data was performed using the Vanquish UHPLC (ThermoFisher, Germany) coupled with an Orbitrap Q Exactive HF-X mass spectrometry (ThermoFisher, Germany) in Novogene Co., Ltd. (Beijing, China).The chromatographic column for separation was Hypersil Gold (2.1 × 100 mm, 1.9 μm) using a 17 min linear gradient at a flow rate of 0.2 mL/min. The eluents for the positive polarity mode were eluent A (0.1% FA in Water) and eluent B (Methanol).The eluents for the negative polarity mode were eluent A (5 mM ammonium acetate, pH 9.0) and eluent B (Methanol).The gradient elution programme was as follows: 0–1.5 min, 2% B; 1.5–12 min, 2–100% B; 12–14 min, 100% B; 14–14.1 min, 100–2% B; 14.1–17 min, 2% B. The data acquisition range was 100–1500 m/z. The mass spectrometry was operated in positive/negative polarity mode with spray voltage of 3.2 kV, capillary temperature of 320 °C, sheath gas flow rate of 40 arb and aux gas flow rate of 10 arb, Funnel RF level of 40, Aux gas heater temperature of 350 °C.

All MS data, including retention time, m/z, and ion intensity, were also aligned and normalized by the Compound Discoverer 3.1 (CD3.1, ThermoFisher). Peaks were aligned with a retention time tolerance of 0.2 min and an actual mass tolerance of 5 ppm. Data were collected using a signal intensity tolerance of 30%, a signal/noise ratio of 3, and minimum intensity. After that, peak intensities were normalized to the tot al spectral intensity [38]. Metabolites were identified by matching with the mzCloud (https://www.mzcloud.org/), mzVault, and MassList databases.The multivariate data matrix was analysed with SIMCA-P^+^ (version 14.1, Umetrics, Umeå, Sweden), and KEGG-enriched metabolic pathways of differential metabolites was analysed with online tool MetaboAnalyst 5.0.

### HPLC analysis of 1′,4′-*trans*-ABA-diol

Single conidia of the mutants and their control strains was cultured on solid PDA medium for 8 days at 25 °C. The 1′,4′-*trans*-ABA-diol in the PDA medium agar block was extracted with 1 mL of acetone for 24 h. After complete drying away from light, it was added to 1 mL of mobile phase and dissolved, and filtered through a 0.22-μm pore-size MCE syringe filter unit. The fermentation broth was centrifuged at 10,000 rpm for 3 min, and the supernatant filtered through a 0.22-μm pore-size nylon syringe filter unit. All sample were measured with high performance liquid chromatography (HPLC) using laboratory prepared samples (98%, w/w) as the standard sample. The HPLC equipment consisted of Agilent G1311C pump, a Agilent G1329B injector volume set 10 μL and a Agilent G1314F detector set 254 nm. Analyses were performed in a YMC-Carotenoid column(4.6 × 250 mm,tosoh,Tokyo,japan) at temperature 37 ℃ with a mobile phase of methanol/water(70:30,v/v) at a flow rate of 0.8 mL/min [33]. The concentration calculated from standard curve of peak area versus concentration. All measurements were performed independently in triplicate.

### qRT-PCR analysis

The mycelia of mutants and their control strains were collected, quenched in liquid nitrogen, and ground into powder immediately. Total RNA was extracted by E.Z.N.A.™ Fungal RNA Kit (OMEGA,USA). RNA integrity was observed by agarose gel electrophoresis, and concentration was determined by NanoDrop spectrophotometer (Thermo Fisher Scientific, Waltham, MA, USA).The synthesis of cDNA was carried out by taking 1 μg of total RNA with PrimeScript ™ RT reagent Kit with gDNA Eraser (Takara Bio, Japan).All cDNA products were diluted tenfold prior to use. The reaction system was designed and amplified according to the instructions of the AceQ Universal SYBR qPCR Master Mix (Vazyme, Nanjing, China). The actin gene (Bcin16g02020) was used to correct for sample-to-sample variation in the amount of RNA. The fold change in the mRNA was calculated by the 2^−ΔΔCt^ method.

### Statistical analysis

Date from control and other sample were processed using SPSS 22.0 software for Windows (SPSS, Chicago, IL, USA). A one-way ANOVA of Tukey analysis was carried out with a confidence interval of 95%, and statistical significance was considered if the *P*-value < 0.05.

## Results and discussion

### Construction of strain ZX2 for the synthesis of 1′,4′-*trans*-ABA-diol

**Fig. 2:**
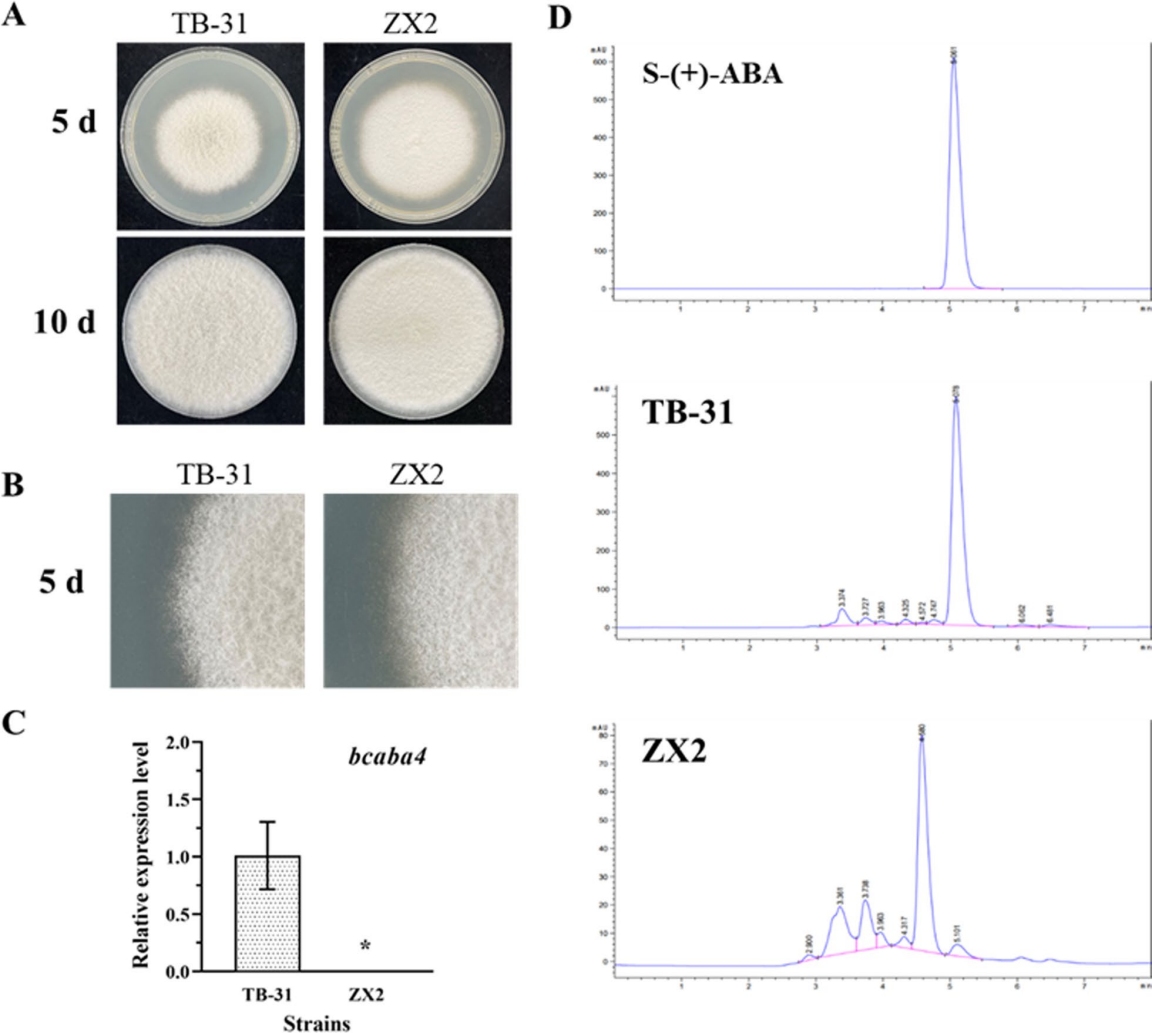
Identification of ZX2 strains and metabolite detection. **A** Phenotype of parental strain *B. cinerea* TB-31 and mutant ZX2 inoculated on PDA for 5 days and 10 days. **B** The mycelial morphology of *B. cinerea* TB-31 and mutant ZX2 on PDA media for 5 days. **C** RT-qPCR examining the transcription level of *bcaba4*. **D** Productivity of *B. cinerea* TB-31 and mutant ZX2 grown on PDA for 8 days, and a commercial S-( +)-ABA was used as the standard sample

We constructed the knockout transformant, the conserved domain was replaced by hygromycin expression cassette, and diagnostic PCR and qRT-PCR was used to confirm the deletion of *bcaba4* gene in ΔBcaba4 transformant (Fig. S1). We conducted an investigation on the phenotype of strain ZX2 and observed that it exhibited a faster growth rate, a dense and fluffy mycelium, and more regular and flat colony edges compared to strain TB-31. Additionally, HPLC analysis showed a shift in the metabolite response peaks of strain ZX2, indicating a change in the synthesized metabolites compared to the ABA response peaks of strain TB-31(Fig. 2). It is worth noting that traces of ABA were still present in the metabolites of strain ZX2 (Fig. 2D), which is consistent with previous studies in where the *bcaba4* gene was knocked out in *B. cinerea* [20]. This is due to the non-enzymatic oxidation that can occur at C-4, resulting in the presence of ABA in the ΔBcaba4 mutant [39].

**Fig. 3:**
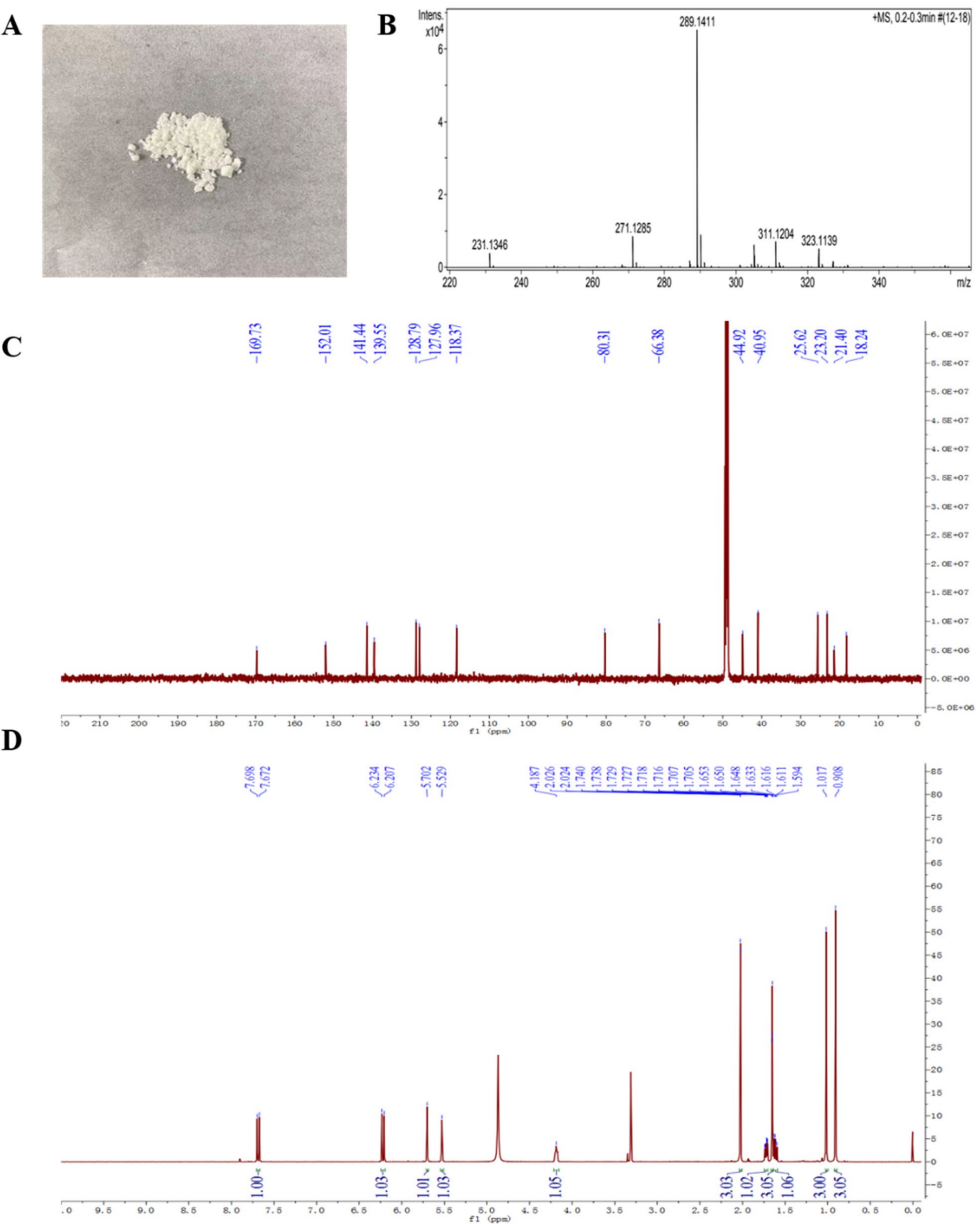
**A** The morphology of 1′,4′-*trans*-ABA-diol; **B** Mass spectrum of 1′,4′-*trans*-ABA-diol. **C** ^1^H NMR spectrum of 1′,4′-*trans*-ABA-diol (600 MHz, CD_3_OD). **D** ^13^C NMR spectrum of 1′,4′-*trans*-ABA-diol (150 MHz, CD_3_OD)

According to the method of wang et al. we concentrated the ZX2 fermentation broth and separated it by ethyl acetate extraction [29]. Its physical and chemical properties and spectral data were as follows: white powder, easily soluble in methanol, molecular formula C_15_H_22_O_4_. HRESIMS *m/z* 289.1411 [M + Na]^+^. HRESIMS *m*/*z* 289.1411 [M + Na]^+^. ^1^H NMR (CD_3_OD, 600 MHz):* δ*_H_ 7.69 (1H, d, *J* = 15.6 Hz, H-5), 6.22 (1H, d, *J* = 15.6 Hz, H-4), 5.70 (1H, s, H-2), 5.53 (1H, s, H-3′), 4.13 (1H, m, H-4′), 2.02 (3H, d, *J* = 1.2 Hz, H-6), 1.72 (1H, ddd, *J* = 13.2, 6.6, 1.2 Hz, H-5′α), 1.65 (3H, d, *J* = 1.8 Hz, H-7′), 1.62 (1H, dd, *J* = 13.2, 10.2 Hz, H-5'β), 1.02 (3H, s, H-8′), 0.91 (3H, s, H-9′); ^13^C NMR (CD_3_OD, 150 MHz): *δ*_C_ 169.7 (C-1), 152.0 (C-3), 141.4 (C-4), 139.5 (C-2′), 128.7 (C-3′), 127.9 (C-5), 118.3 (C-2), 80.3 (C-1′), 66.3 (C-4′), 44.9 (C-5′), 40.9 (C-6′), 25.6 (C-9′), 23.2 (C-8′), 21.4 (C-6), 18.2 (C-7′) (Fig. 3). The structure of this substance is similar to ABA, and it has been reported to be a precursor substance for the synthesis of ABA [40].

**Fig. 4:**
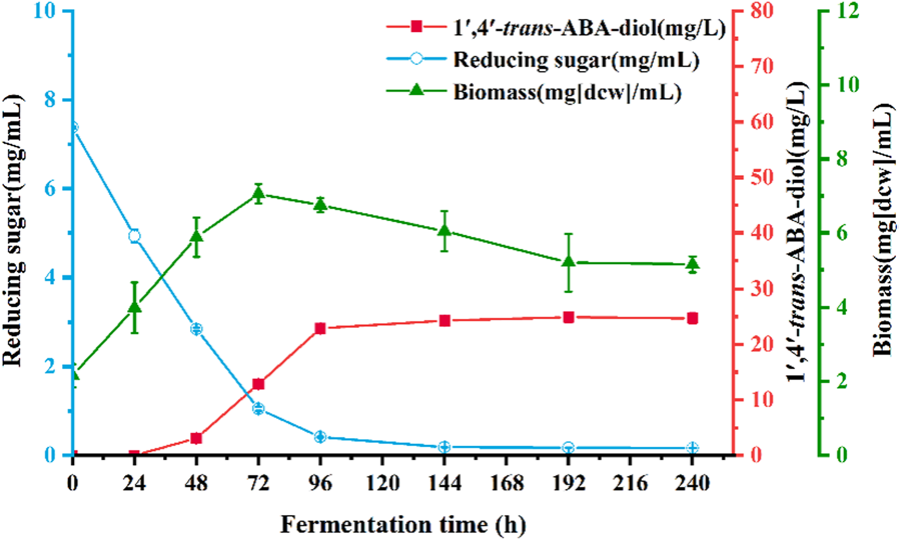
The batch fermentation of 1′,4′-*trans*-ABA-diol from *B. cinerea* ZX2 with time in shake flasks. All values presented are the mean of three biological replicates ± standard deviation

After confirming that the product of strain ZX2 was 1′,4′-*trans*-ABA-diol, we tested the metabolism of this strain during a10-day shake flask fermentation (Fig. 4). As shaking began, the concentration of reducing sugars gradually decreased until 144 h. The biomass initially increased (0–72 h) and then gradually declined until the end of fermentation. The production of 1′,4′-*trans*-ABA-diol continued to increase (24–192 h), and reaching its maximum concentration of 4.79 mg/g DCW(24.8 mg/L) at 192 h.

### Fed-batch fermentation of *B. cinerea* ZX2 for 1′,4′-*trans*-ABA-diol

**Fig. 5:**
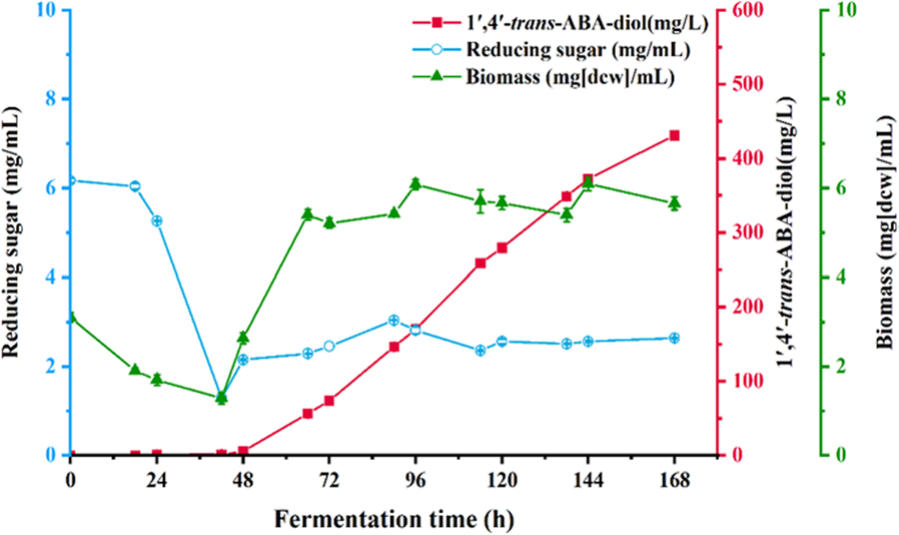
Fed-batch fermentation curve for 50 L fermenter. Seed culture was incubated at 25 °C and 180 rpm for 40 h. The fermentation condition of fed-batch fermentation in the fermenter was performed at 23–26 °C, 200–400 rpm and with 10–70% dissolved oxygen. 5% glucose solution was continuously fed into the fermenter after 72 h. All values presented are the mean of three biological replicates ± standard deviation

To investigate the growth and fermentation performance of the ZX2 strain, a 50 L fed-batch fermentation process was conducted in a fermenter (Fig. 5). Over the course of the fermentation, the concentration of reducing sugars decreased and eventually stabilized at 2.0 mg/mL after 48 h. This level was maintained through the addition of sucrose solution. At the same time, the synthesis and accumulation of 1′,4′-*trans*-ABA-diol began at 42 h and gradually increased throughout the fermentation process, reaching a concentration of 76.1 mg/g DCW (430.9 ± 0.7 mg/L) at the end of the 168-h fermentation. Based on the 1′,4′-*trans*-ABA-diol change rule, the fermentation process of the ZX2 strain can be divided into two stages. During the first 42 h of fermentation, the strain primarily undergoes cell growth and accumulates precursors for the product. After 42 h, there is a significant increase in the synthesis of the product, 1′,4′-*trans*-ABA-diol. Based on the synthesis of product, the fermentation process can be divided into two stages: cell growth and product synthesis. In the first 48 h of fermentation, the strain is in the growth stage, continuously consuming carbon sources, nitrogen sources, phosphates, and other substances for cell synthesis. During this stage, the cells undergo slow proliferation. After this, the strain enters the product synthesis stage and begins to rapidly increase in biomass as it synthesizes 1′,4′-*trans*-ABA-diol. After 72 h of fermentation, the biomass of the strain remains stable and the rate of product synthesis reaches its maximum.

### Metabolomic analysis of fermentation broth metabolites from *B. cinerea* ZX2

**Fig. 6:**
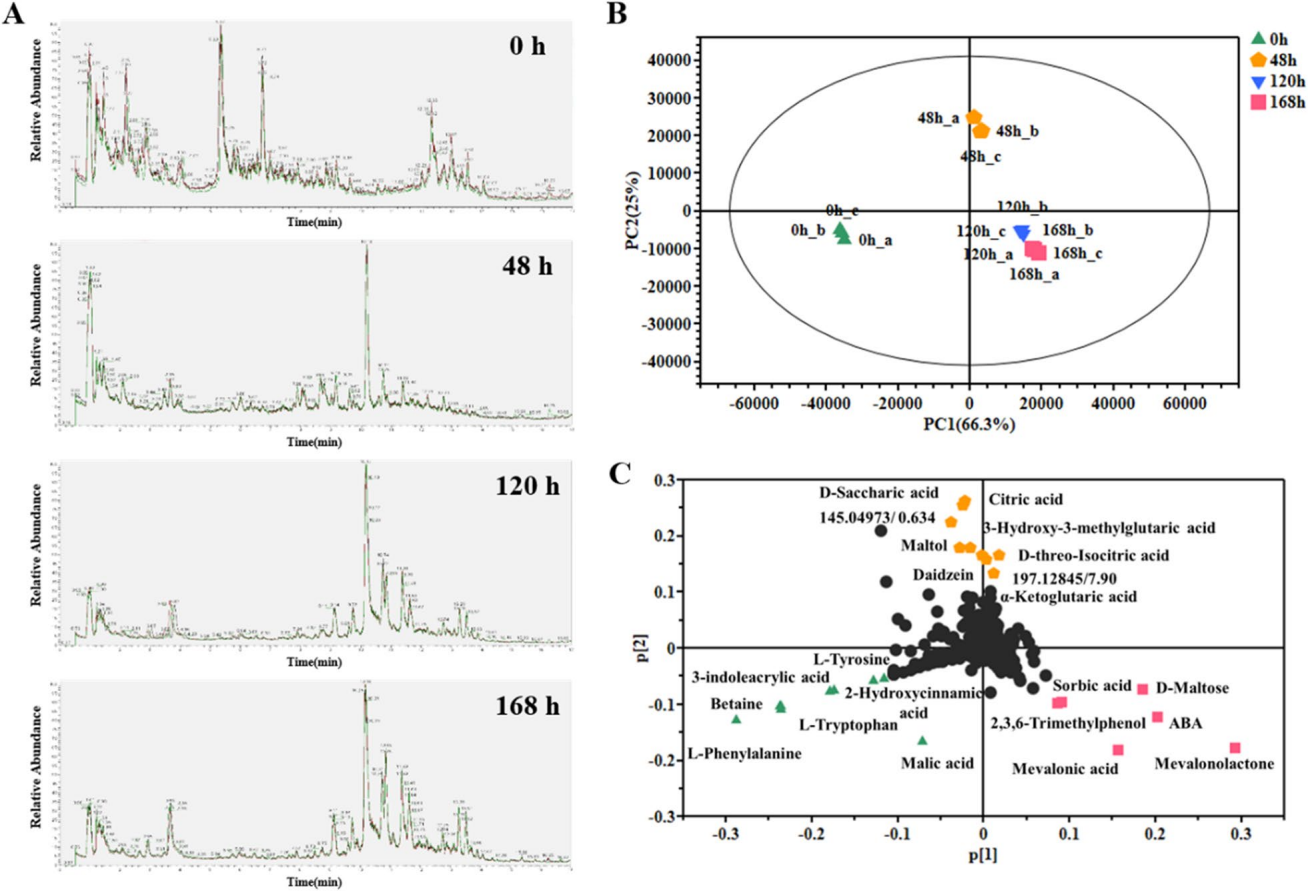
**A** Total Ion Chromatogram (TIC) of different periods of broth. The mass spectrometry was operated in ESI positive mode. **B** PCA score plots of different periods of broth in total ion mode. **C** PCA loading plots of different periods of broth. Three samples were analyzed from each time point

Samples of fermentation broth were collected and analyzed, resulting in the identification of 470 metabolites in negative ion mode and 1075 metabolites in positive ion mode. The abundance of some metabolites changed significantly during the fermentation process. There was little difference between the 120 h and 168 h samples, with the main compounds being produced at 48 h into fermentation (Fig. 6A). The PCA score plot based on the extracted ion chromatogram of the broth clearly showed distinct clustering for each time point (0, 48, 120, and 168 h), with each group being easily distinguishable from the others (Fig. 6B). The late stage-fermented broth (120 and 168 h) were clearly differentiated from the early-stage fermented broth (0 and 48 h) by principal component 1, while the 0- and 48-h fermented broth samples were clearly distinguished from the non-fermented broth (0 days) by the principal component 2. The PCA loading plot illustrates the potential marker compounds that contributed to the differences between the various broth samples (Fig. 6C). During fermentation, L-phenylalanine, betaine, 3-indoleacrylic acid, L-tryptophan, L-tyrosine, 2-hydroxycinnamic acid, malic acid, mevalonolactone, mevalonic acid, D-maltose, sorbic acid, 2,3,6-trimethylphenol, ABA, citric acid, D-saccharic acid, maltol, daidzein, 3-hydroxy-3-methylglutaric acid, α-ketoglutaric acid, and two unidentified compounds away from the origin of the axes, which may have a significant effect on the fermentation process. Regarding the appearance of ABA, our hypothesis is that non-enzymatic oxidation of 1ʹ,4ʹ-*trans*-ABA-diol leads to the microsynthesis of ABA. This is in accordance with the results of our HPLC analysis.

Additionally, the OPLS-DA models were able to clearly distinguish between each fermentation step (0–48, 48–120 and 120–168 h) (Fig. S2). The quality parameters of the models, including high-fitting (*R*^*2*^ ≥ 0.993) and high-predicting (*Q*^*2*^ ≥ 0.949) qualities, were validated by a 200 times permutation test. Based on VIP ≥ 2 and *P* < 0.05, 107 out of 1545 compounds showed significant differences in abundance among the fermented samples at 0, 48,120 and 168 h (Fig. S2). The appearance and accumulation of metabolites in fermentation broths are of particular interest. Therefore, we investigated the differential metabolite profile that was up-regulated during fermentation (Table 3). During the fermentation process, the level of TCA cycle substances citrate and α-ketoglutaric acid initially increased and then decreased, while malic acid showed a decrease, followed by an increase, and then a final decrease. The compounds 3-hydroxy-3-methylglutarate, mevalonolactone, and mevalonate in the MVA pathway showed an increasing trend. By-products such as sorbic acid, resorcinol, hydroquinone, and 3-isopropylmalonic acid also accumulated throughout the fermentation process. Accumulation of metabolites in the MVA pathway during fermentation suggests the presence of bottleneck points that may hinder the metabolic flux of 1′,4′-*trans*-ABA-diol. For example, HMGR and ERG12 are all key enzymes in the MVA pathway [41, 42].Therefore, there is a necessity for metabolic engineering to alleviate these bottleneck points in the 1′,4′-*trans*-ABA-diol biosynthesis pathway and ultimately improve the synthetic capacity of the strain.

**Table 3:** Relative quantification of up-regulated metabolites

Metabolite no	Differential metabolite	Fold change (48/0 h)	p-value (48/0 h)	Fold change (120/48 h)	p-value (120/48 h)	Fold change (168/120 h)	p-value (168/120 h)
1	4-Hydroxybutyric acid (GHB)	**8.54**	< 0.01	0.19	< 0.01	0.90	0.22
2	D-threo-Isocitric acid	**5.54**	< 0.01	0.28	< 0.01	0.95	0.70
3	alpha-Ketoglutaric acid	**4.97**	< 0.01	0.26	< 0.01	0.97	0.59
4	2,5-Furandicarboxylic acid	**4.96**	< 0.01	0.27	< 0.01	0.89	0.44
5	Daidzein	**4.18**	< 0.01	0.26	< 0.01	0.67	< 0.01
6	3-(propan-2-yl)-octahydropyrrolo[1,2-a]pyrazine-1,4-dione	**2.33**	< 0.01	0.43	< 0.01	0.77	0.03
7	Citric acid	**2.73**	< 0.01	0.23	< 0.01	0.81	0.08
8	D-Saccharic acid	**2.72**	< 0.01	0.22	< 0.01	0.81	0.07
9	3-Hydroxy-3-methylglutaric acid	**1.86**	0.01	0.47	< 0.01	0.77	0.13
10	Abscisic acid	**221.35**	< 0.01	**3.04**	< 0.01	0.84	0.02
11	2,3,6-Trimethylphenol	**121.96**	< 0.01	**6.76**	< 0.01	1.15	0.08
12	1-[4-hydroxy-3-(3-methylbut-2-en-1-yl)phenyl]ethan-1-one	**55.74**	< 0.01	**3.29**	< 0.01	0.88	0.04
13	Mevalonolactone	**22.82**	< 0.01	**1.98**	< 0.01	1.43	< 0.01
14	L-Iditol	**3.29**	< 0.01	**2.96**	< 0.01	0.50	< 0.01
15	D-(-)-Mannitol	**2.30**	< 0.01	**1.76**	< 0.01	0.33	< 0.01
16	9-HpOTrE	0.16	< 0.01	**7.32**	< 0.01	1.06	0.53
17	Malic acid	0.14	< 0.01	**4.08**	< 0.01	1.13	0.20
18	Avocadyne 1-acetate	0.57	0.01	**13.56**	0.02	**2.29**	0.03
19	Palmitic acid	1.28	0.04	0.49	< 0.01	**4.38**	0.36
20	Elaidic acid					**3.16**	0.02
21	Sorbic acid	**9.41**	< 0.01	**5.77**	< 0.01	**1.63**	< 0.01
22	Mevalonic acid	**3.94**	< 0.01	**4.94**	< 0.01	**1.72**	< 0.01
23	3-Isopropylmalic acid	**3.32**	< 0.01	**2.19**	0.03	**3.87**	< 0.01
24	Resorcinol	**1.78**	< 0.01	**28.95**	< 0.01	**2.90**	< 0.01
25	Hydroquinone	**1.65**	< 0.01	**5.98**	< 0.01	**2.62**	< 0.01

Bold indicates positive fold change > 1.5

### Transcriptional analysis of the genes for 1′,4′-*trans*-ABA-diol synthesis in *B. cinerea* ZX2

**Fig. 7:**
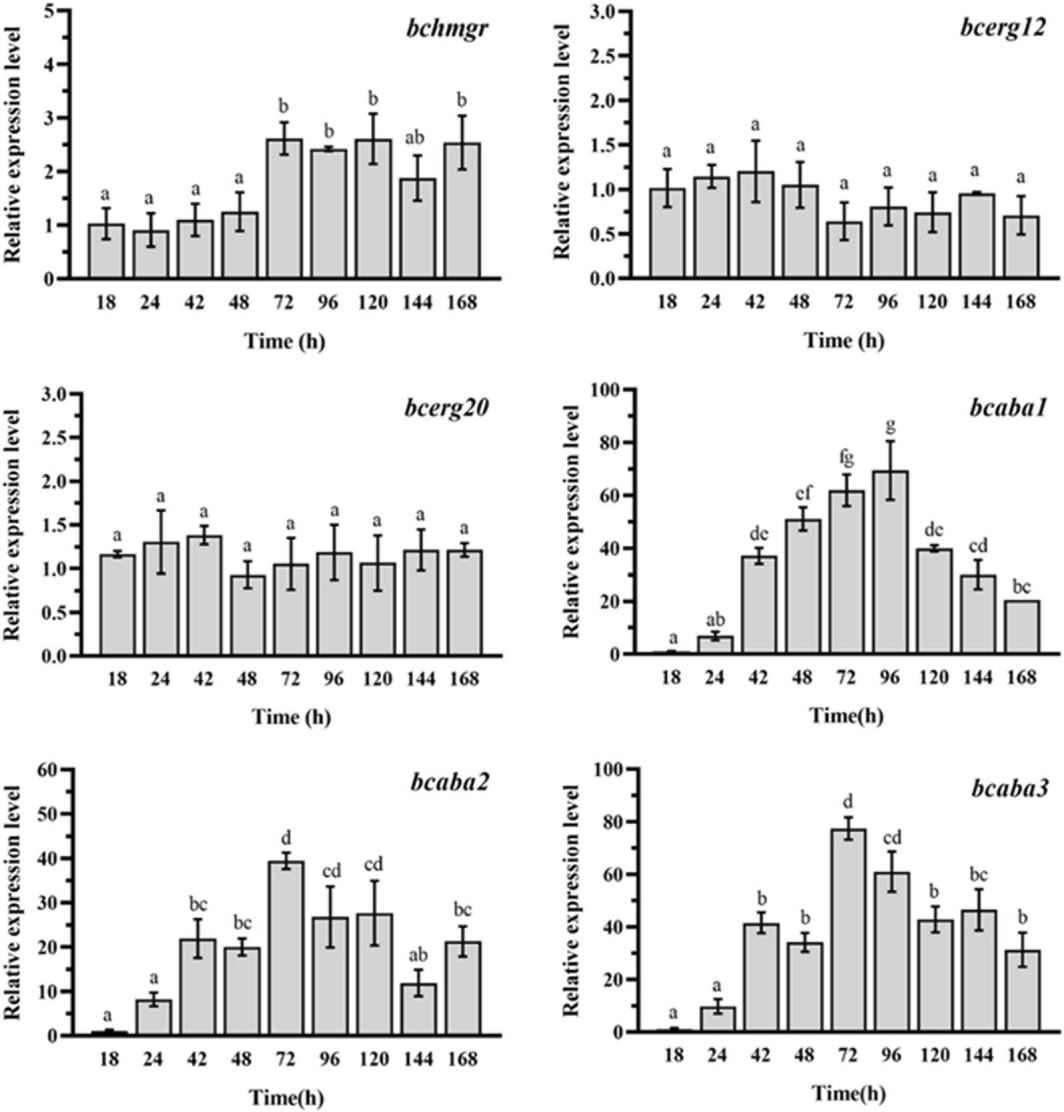
Expression models of key genes during 50 L fed-batch fermentation. Different letters represent significant differences in relative expression between groups (*P* < 0.05)

In the *B. cinerea* ZX2 strain, 1′,4′-*trans*-ABA-diol is synthesized through the MVA pathway and a partial cluster of ABA-synthesis genes. However, the expression of these genes during fermentation is unknown. To investigate this, we utilized strain ZX2 to produce 1′,4′-*trans*-ABA-diol in a 50-L fermenter and monitored the transcriptional expression levels of the genes throughout the fermentation process. Hydroxy-3-methylglutaryl reductase (*bchmgr*) and mevalonate kinase (*bcerg12*) affect the reaction rate of the MVA pathway [43]. The HMG-CoA reductase (HMGR) enzyme is responsible for converting 3-hydroxy-3-methylglutaryl CoA to mevalonate, which is the only redox cofactor utilization step in the entire MVA pathway and a crucial bottleneck in the catalytic reaction. Mevalonate kinase (ERG12) catalyzes the conversion of mevalonate to mevalonate-5-phosphate, the first phosphorylation reaction in the MVA pathway. Farnesyl pyrophosphate synthase (ERG20) is the first branching point of the MVA pathway. FPP is a fundamental substrate for many biomaterials, and in *Saccharomyces cerevisiae* the size of the FPP pool is a determining factor in the production of sesquiterpenes [44]. Previous studies have shown that GPP, FPP and geranylgeranyl diphosphate (GGPP) can inhibit ERG12 activity [45]. Since FPP, GPP, and GGPP are important precursors for terpenoid synthesis, ERG12 plays a crucial role in regulating terpenoid biosynthesis. Analysis of gene transcript levels revealed that the *bchmgr* gene was up-regulated by approximately twofold at 72 h, while there was no significant change in the expression of the *bcerg12* and *bcerg20* genes (Fig. 7).

Additionally, we examined the transcript levels of the *bcaba1-3* genes in the ABA synthesis gene cluster, which exhibited a more than tenfold increase in expression at the onset of product synthesis (42 h).This high level of expression was maintained until the end of the fermentation. These results suggest that changes in the expression of *bchmgr*, *bcerg20*, and *bcaba1-3* during 1′,4′-*trans*-ABA-diol fermentation are closely linked to product synthesis(see Fig.S5 for the rest of the genes).

### Construction of Vector pCBG418 Suitable for the ATMT of *B. cinerea* ZX2

Although strain ZX2 was able to stably synthesize 1′,4′-*trans*-ABA-diols through shake flask fermentation, the yield was not optimal. Analysis of metabolomic data and transcript expression of 1′,4′-*trans*-ABA-diol synthesis genes revealed that there was an overflow of precursors during fermentation and that the expression of certain synthesis genes was strongly correlated with yield. To address this issue, we implemented a static regulatory strategy by introducing additional copies of genes encoding key enzymes into the genome. This was done in order to increase the carbon flux of the synthetic pathway and thus improve the efficiency of 1′,4′-*trans*-ABA-diol synthesis. However, filamentous fungi have a limited number of available screening markers, and not all of them are effective [46]. In the case of the ZX2 strain, the *bcaba4* gene was replaced with a resistance gene *hph* expression cassette, which eliminated its susceptibility to Hygromycin. Additionally, we found that *B. cinerea* is not highly sensitive to glufosinate, and using it as a screening marker resulted in a high false-positive rate, which was not helpful for the sub-culture of the modified strain (data not shown). To determine the most suitable screening marker, we tested the susceptibility of the ZX2 strain to four different antibiotics: Bleomycin (BLM), Geneticin (G418), Nourseothricin (NTC), and Benomyl (Fig. S4). We found that the ZX2 strain was able to grow on PDAs containing BLM at concentration ranging from 20 to 320 μg/mL. However, colony growth was inhibited at concentrations of 0.2 µg/ml for Benomyl, 160 µg/ml for NTC, and 160 µg/ml for G418. While Benomyl is effective at low concentrations, it needs to be dissolved in acetone, which we found to have a detrimental effect on the growth of the strains at a concentration of 4 µL/mL)., Additionally, NTC is expensive (approximately 10–15 times more expensive than G418), so we ultimately decided to a concentration of 160 μg/mL of G418 as the screening condition. G418 is an aminoglycoside antibiotic that interferes with 80S ribosome function in eukaryotic cells and is commonly used as a screening marker for eukaryotic cells. Its cross-resistance gene, *neo*, encodes neomycin phosphotransferase II, which catalyzes the phosphorylation and then inactivation of aminoglycoside antibiotics such as Geneticin G418, Neomycin and Kanamycin [47]. The binary vector pCBg418 of size 9608 bp suitable for this strain of ATMT, was constructed based on the results of sensitivity testing.

### Overexpression mutants and genetic stability

Six overexpression plasmids, pCBHR, pCBMK, pCBFS, pCBA1, pCBA2, and pCBA3, were obtained by recombining the cDNAs of *bchmgr*, *bcerg12*, *bcerg20*, *bcaba1*, *bcaba2*, and *bcaba3* with linearized pCBg418, respectively. These plasmids were then used for ATMT of *B. cinerea* ZX2. The resulting resistant transformants were confirmed by PCR using primers test-F/test-R to ensure that the target gene was successfully inserted in the MCS region was integrated into the genome of these mutants. Each mutant was then cultured for at least three consecutive generations of single spore isolation on PDA plates containing both antibiotics. After this, the mutant strains were grown on PDA plates for 8 days, and the level of 1′,4′-*trans*-ABA-diols were determined using agar block method. Additionally, a subset of randomly selected mutant strains were examined using qRT-PCR to measure the expression levels of the target mRNA (Fig S6). The results showed all the overexpression mutants exhibited a significant increase of more than twofold in gene expression levels compared to the parental ZX2 strain, indicating successful overexpression of the integrated genes were overexpressed.

**Fig. 8:**
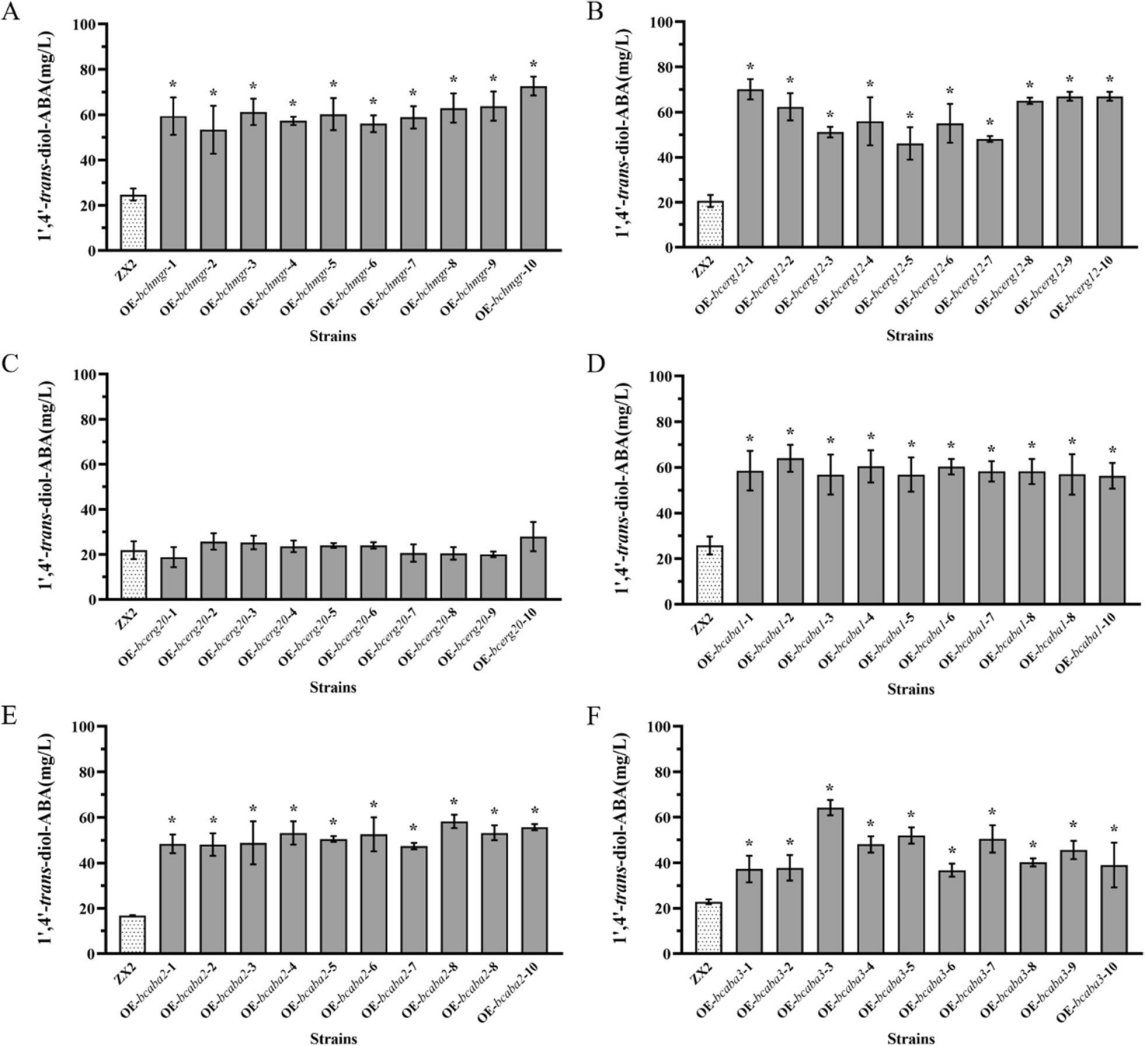
Effects of single-gene overexpression of genes involved in the MVA pathway on 1′, 4′-*trans*-ABA-diol production. **A** OE *bchmgr* strain; **B** OE *bcerg12* strain; **C** OE *bcerg20* strain; **D** OE *bcaba1* strain; **E** OE *bcaba2* strain; **F** OE *bcaba3* strain. *Represents *p*-value < 0.05 compared to ZX2

The OE-*bchmgr* strain exhibited a significantly higher yield, with the maximum concentration of 1′,4′-*trans*-ABA-diol reaching 72.6 mg/L. This was also observed in the OE-*bcerg12* strain, with a peak concentration of 70.1 mg/L. These findings are consistent with previous studies on increasing terpenoid production. In *S. cerevisiae*, co-overexpression of tHMGR1 (truncated HMGR1), ERG12, and IDI1^WWW^ genes in the MVA pathway resulted in a 62.31 mg/L production of the D-limonene to [43]. This is attributed to the overexpression of the rate-limiting enzyme HMGR, which down-regulates competitive metabolic ways and increase the supply of acetyl coenzyme A in the cytoplasm [48]. The yield of 1´, 4´-*trans*-ABA-diol remained unchanged in the OE-*bcerg20* strain. In *Yarrowia lipolytica,* Yang et al. reported a 20.8-fold increase in α-farnesene produced by co-overexpressing *tHMG1*, *IDI*, and *ERG20* genes [49]. Although heterologous sesquiterpene synthase genes in yeast can produce corresponding sesquiterpenes, the precursor supply is often insufficient, and a simultaneous overexpression of ERG20 and related genes is necessary to enhance the yield [50, 51]. In the ZX2 strain, there was a sufficient supply of precursors, and the enzymatic steps involved in ERG20 may not be critical for efficient synthesis, thus overexpression of the *bcerg20* did not improve yields. The highest concentrations of 1′,4′-*trans*-ABA-diol were 64.0 mg/L, 58.2 mg/L, and 64.2 mg/L for the OE-*bcaba1*, OE-*bcaba2*, and OE-*bcaba3* strains, respectively. These results were consistent with previous studies. In *S. cerevisiae *CEN.PK113-5D, overexpression of *bcaba1* alone doubled ABA production, while overexpression of *bcaba2* and *bcaba3* also slightly increased ABA titre [22]. Similarly, in the ABA-producing *Y. lipolytica*ST9726, the addition of two copies of *bcaba1* increased the yield by 5% and the addition of one copy of *bcaba3* increased the yield by 15% [52]. In our study, we found that overexpression of key genes to enhance carbon flux through the MVA pathway is an effective strategy for increasing 1′,4′-*trans*-ABA-diol production in *B. cinerea*. In contrast, the control strain TB-31, which carried only the pCBg418 empty vector, did not show an increase in ABA production (data not shown). Therefore, we conclude that overexpression of *bchmgr*, *bcerg12*, *bcaba1*, *bcaba2*, and *bcaba3* in the ZX2 strain positively impacted the synthesis of 1′,4′-*trans*-ABA-diol (see Fig. 8).

### Genetic stability studies and shake flask fermentation validation

**Fig. 9:**
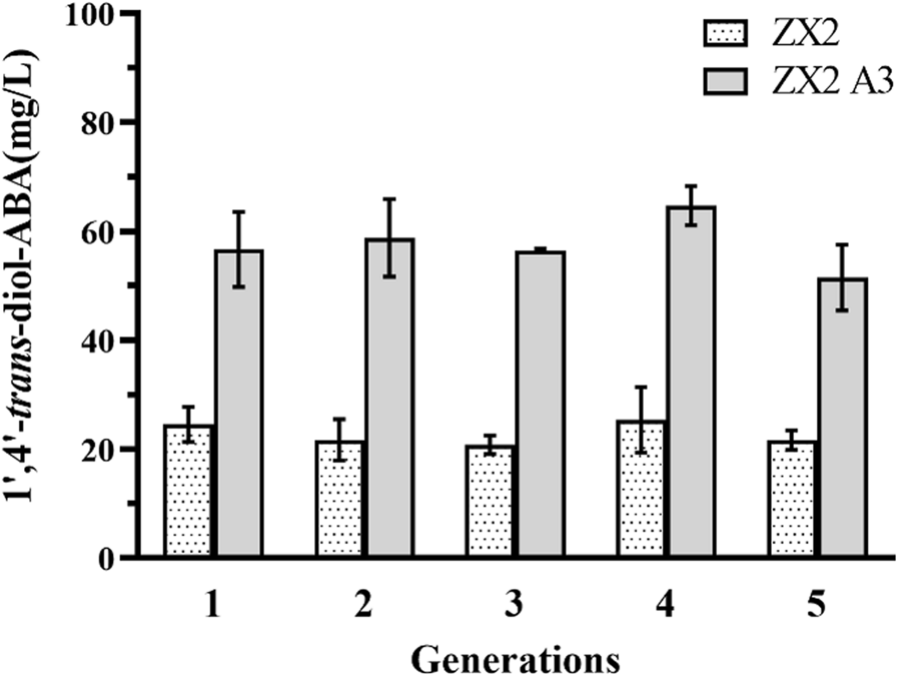
Each generation of overexpressing strains was cultured on PDA media for 7 days and the yield of 1′,4′-*trans*-ABA-diol was determined

Compared to unicellular microorganisms such as *E. coli* and *S. cerevisiae*, filamentous fungi have complex genetic characteristics and are prone to the formation of heterokaryons after genetic engineering modifications, which leads to trait instability [53]. To tackle this issue, we cultured the high-yielding mutants for five successive single spore isolation and determined the 1′,4′-*trans*-ABA-diol yield of each generation using the agar block method. The results indicate that certain mutant strains exhibited regressive mutations in the subsequent generation, resulting in decreased production of 1′,4′-*trans*-ABA-diol. However, we were fortunate to discover ZX2 A3 (strain 3 in the OE-*bcaba3* group of strains), which displayed consistent and high levels of production. After five split cultures, there was no decrease in yield, indicating a stable high-yielding trait (Fig. 9).

**Fig. 10:**
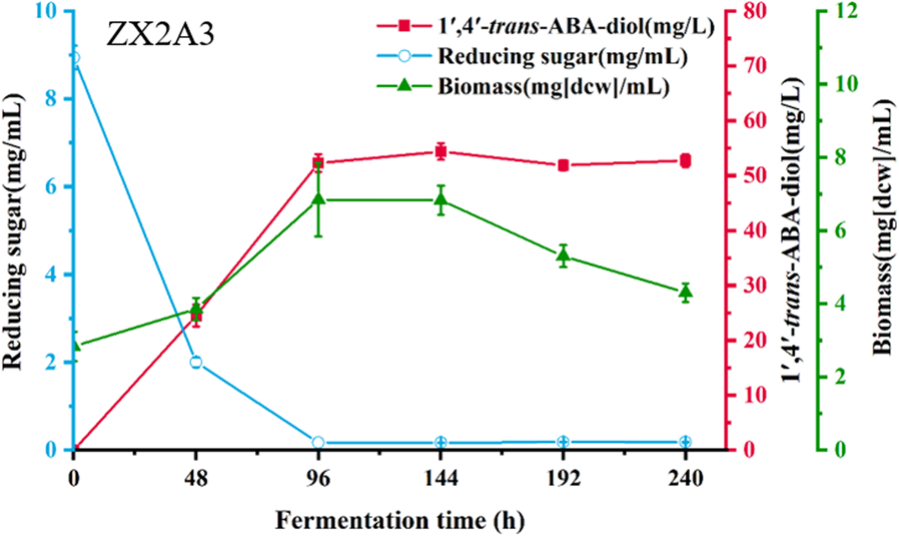
Time course of shake flask fermentation of 1′,4′-*trans*-ABA-diol in overexpression strain ZX2-A3.The cells of ZX2 A3 was cultivated at 25 °C and 200 rpm with 50 mL of liquid fermentation medium in a 250-mL shake flask, and product yield were determined at the 0-, 24-, 48-, 72-, 92-,120- and 144-h time-points. All values presented are the mean of three biological replicates ± standard deviation

To confirm the enhanced ability of the ZX2 A3 strain to synthesize 1′,4′-*trans*-ABA-diol, we conducted shake flask fermentation. The batch fermentation was carried out by inoculating 10% (V/V) of the strain in 50 mL fermentation medium for 10 days, with strain ZX2 as the control (Fig. 10). The reducing sugar concentration of strain ZX2 A3 decreased gradually during the first 96 h of fermentation and remained constant thereafter. The biomass increased gradually during the first 96 h of fermentation, reaching a maximum value of 6.8 g/L, and then declined after 144 h. Synthesis of 1′,4′-*trans*-ABA-diol started at 48 h and reached a maximum value of 7.96 mg/g DCW (54.4 mg/L) at 144 h. In the same conditions, the 1′,4′-*trans*-ABA-diol yield of the overexpression mutant strain was about 2.1-fold higher than that of the ZX2 strain. Shake flask fermentation confirmed the high productivity of the ZX2 A3 strain, and a high yielding strain was obtained.

## Conclusions

Sesquiterpenoids are naturally occurring compounds found in a wide range of plants, and they play a crucial role in both plant growth and human health. These compounds have various applications in agriculture, industry, and medicine, including the use of ABA to regulate plant growth, nerolidol for essential oils, farnesene for clean fuels, and artemisinin used for the treatment of malaria [54]. As the demand for sesquiterpenes continues to grow, there is a growing interest in using microbial fermentation to produce these compounds from renewable raw materials. ABA, for example, there have been several studies on its heterologous cellular biosynthesis. Otto et al. Successfully expressed the *bcaba1-4* genes in *S. cerevisiae*, resulting in a synthetic pathway that produced 11 mg/L of ABA [22]. Similarly, Arnesen et al. increased the copy number of genes involved in the mevalonate pathway and ABA biosynthesis in *Y. lipolytica,* resulting in an impressive ABA yield of 263.5 mg/L [52]. These studies mainly focus on the biosynthesis of taget products in well- characterized model microorganisms.

To date, *B. cinerea* still has the highest ABA yield (up to 2 g/L) compared to other cells, suggesting that it may be a more efficient cellular factory for some sesquiterpenes [12]. Previous studies have also shown that producing of heterologous sesquiterpenoids can cause cellular stress [55]. Our speculation was that by knocking down of the *bcaba4* gene in strain TB-31, the major product would shift to 1′,4′-*trans*-ABA-diol, with a yield similar to that of ABA. Interestingly, we observed changes in the morphology and growth rate of the ZX2 strain, as well as altered the ABA of interest, supporting our hypothesis. However, the production of 1′,4′-*trans*-ABA-diol was only 1/5 of the previous ABA production (unpublished data), indicating that the deletion of the *bcaba4* gene may have affected the expression of other genes in the synthetic pathway. This phenomenon has been observed in fungi, where gene clusters and synthetic pathways can have regulatory effects on each other [56, 57]. The metabolomics results of the fermentation broth of strain ZX2 indicated that the activity of certain enzymes between mevalonate and 1′,4′-*trans*-ABA-diol may be insufficient, leading to the accumulation of intermediate products. Analysis of the transcript expression levels of the synthetic pathway genes revealed that the *bchmgr*, *bcerg12* and *bcaba1-3* genes were associated with product synthesis. We used the ATMT method to transform an overexpression plasmid containing the resistance gene *neo*. In filamentous fungi and other multicellular organisms, the nonhomologous end joining (NHEJ) pathway seems to be more dominant than the homologous recombination (HR) pathway. And since suitable insertion sites are not known, random insertion was used for integration into the genome. While ATMT is time-consuming, this method inserts a lower copy number of DNA and reduces the possibility of copy number interference [58].We confirmed that overexpression of *bchmgr*, *bcerg12*, *bcaba1*, *bcaba2*, and *bcaba3 *genes in *B.cinerea, *respectively, increased 1′,4′-*trans*-ABA-diol production by gene expression and product yield. Furthermore, we obtained a high-yielding strain ZX2 A3. In conclusion, our strategy of increasing the expression of genes in the synthetic pathway was successful in increasing the yield of 1′,4′-*trans*-ABA-diol.

To the best of our knowledge, this is the first report on the fermentation of 1′,4′-*trans*-ABA-diol by a filamentous fungus, especially *B. cinerea*. This groundbreaking research has resulted in the development of a high-yielding strain through the implementation of a static conditioning strategy, a crucial step for its potential application in various industries. The findings from this study will serve as a valuable resources for future research on similar sesquiterpene derivatives. Furthermore, this work significantly contributes to the existing library of filamentous fungal fermentation techniques. Moving forward, we plan to explore various engineering strategies in order to further improve the synthesis efficiency of *B. cinerea* ZX2 strain and expand the potential application prospects of 1′,4′-*trans*-ABA-diol.

### Supplementary Information

Additional file 1.

## Data Availability

The authors confirm that they will upload data on the results of this study.
