# Supplementary material for: Engineering strategies for enhanced 1′, 4′-trans-ABA diol production by Botrytis cinerea
Source: Microb Cell Fact. 2024 Jun 26;23:185. doi: 10.1186/s12934-024-02460-8 (PMC11210036; doi:10.1186/s12934-024-02460-8)
Supplement: Supplementary file 1 — Additional file 1. [file 12934_2024_2460_MOESM1_ESM.docx]

SUPPLEMENTARY INFORMATION

Enhanced 1´, 4´-*trans*-diol ABA production by metabolically engineered *B.cinerea* ZX2

Yifan Wang^1, 2^, Dan Shu^1*^, Zhemin Li^1^, Di Luo^1^, Jie Yang^1^, Dongbo Chen^1^, Tianfu Li^1^, Xiaonan Hou^1, 2^, Qi Yang^1, 2^ and Hong Tan^1*^

1. CAS Key Laboratory of Environmental and Applied Microbiology, Environmental Microbiology Key Laboratory of Sichuan Province, Chengdu Institute of Biology, Chinese Academy of Sciences, Chengdu, China

2. University of Chinese Academy of Sciences, Beijing, China

# SUPPLEMENTARY TABLES

**Table S1.** Primers used in PCR

| Primer | Sequence | Description |
| --- | --- | --- |
| bcaba4-5′F(P1) | TGTGATAGCCCCATTTGTTT | Amplification of the upstream fragment of *bcaba4* |
| bcaba4-5′R(P2) | CCCGAATCGGAATGCGGCTCCACACTCCAAACTTCCTTACATCG |  |
| bcaba4-3′F(P3) | CAATAGTAACCATGCATGGTTGCCTAGCTAAAGACTGCCGAAAACT | Amplification of the downstream fragment of *bcaba4* |
| bcaba4-3′R(P4) | CCAGCCAGATTCGTCTCAAA |  |
| hph-F(P5) | TGTGGAGCCGCATTCCGAT | Amplification of the resistance gene *hph* to construct a knockout cassette |
| hph-R(P6) | TAGGCAACCATGCATGGTTAC |  |
| bcaba4-7(P7) | TTTTCCCATTTGGTTTGAGC | Identification of upstream insertion location |
| bcaba4-8(P8) | TCGTGAATCCGTGACGCTGAAT |  |
| bcaba4-9(P9) | CGGCGAAGCAGAAGAATAG | Identification of downstream insertion location |
| bcaba4-10(P10) | TACGCTCCCACATTATCTTG |  |
| bcaba4-11(P11) | ACTTCCTTTGCTGCCGTTCT | Identification of knockout genes |
| bcba4-F(P12) | GAATTAAAGTAAGATATTCCTGAAAGC | Amplification of the ORF for *bcaba4* |
| bcaba4-R(P13) | GGCAGCAATCCACGAGA |  |
| neo-F | TTCTACCCAAGCATCGATATGATTGAACAAGATGGATTGCACG | Amplification of the resistance gene *neo* to construct pCBg418 |
| neo-R | ACATTATTATGGAGAAATCAGAAGAACTCGTCAAGAAGGCGA |  |
| CaMV35S-F | TGACGAGTTCTTCTGATTTCTCCATAATAATGTGTGAGTAGTTCCCAG | Amplification of the terminator to construct pCBg418 |
| CaMV35S-R | CTGAATTAACGCCGAATTGATCTGGATTTTAGTACTGGATTTTGGTTTTAG |  |
| pCBh1-AnoliC | GTCAAACACTGATAGTTTAAACTGAAGGCGGGAAACG | Amplification of MCS cassette and promoter of resistance gene *neo* to construct pCBg418 |
| pCBh1-AntrpC | TCCATCTTGTTCAATCATATCGATGCTTGGGTAGAATAGGT |  |
| HMGR-F | AATCGATCCGAATTCGAGCTCATGATTGGAAATACGCTCCTGC | Amplification of *bchmgr* to construct pCBHR |
| HMGR-R | TGCCTGCAGGTCGACTCTAGACTATTTCTTTCCCCCTGCAGC |  |
| MK-F | AATCGATCCGAATTCGAGCTCATGCCTGTGCGTGACAGCA | Amplification of *bcerg12* to construct pCBMK |
| MK-R | TGCCTGCAGGTCGACTCTAGACTAATCATCTTCAACCCTCCAAAAC |  |
| FPPS-F | AATCGATCCGAATTCGAGCTCATGGCGAAGGCTACCACTCTC | Amplification of *bcerg20* to construct pCBFS |
| FPPS-R | TGCCTGCAGGTCGACTCTAGATTACTTGCTTCTCTTGTAAATCTTGCT |  |
| ABA1-F | AATCGATCCGAATTCGAGCTCATGTCTAATTCTATATTGAACCTAGGCTCC | Amplification of *bcaba1* to construct pCBA1 |
| ABA1-R | TGCCTGCAGGTCGACTCTAGACTATTTGTATTCTGTTCCCTCACTTTCA |  |
| ABA2-F | AATCGATCCGAATTCGAGCTCATGCTGCTTAGCATTAAAGACCTG | Amplification of *bcaba2* to construct pCBA2 |
| ABA2-R | TGCCTGCAGGTCGACTCTAGACTATCTAGGAACCTCTTTTAACATGACC |  |
| ABA3-F | AATCGATCCGAATTCGAGCTCATGCAGCAAGTTATTACTCAAACG | Amplification of *bcaba3* to construct pCBA3 |
| ABA3-R | TGCCTGCAGGTCGACTCTAGATCAAACTGGAACCTCAAAATGTG |  |
| test-F | CCCATTTTTCTTCGGTTCC | Identify clones and sequence plasmids |
| test-R | TGTATCTGGAAGAGGTAAACCC |  |

**The underlined sequences indicated the homologous oligonucleotides that facilitate In-Fusion Assembly reactions.**

**Table S2.** List of primers used for the quantitative PCR analysis.

| **Gene** | **GenBank**  **accession number** | **Primer** | **Sequence** | **size(bp)** | **Slope** | **Amplification efficiency (%)** | **(R^2^)** |
| --- | --- | --- | --- | --- | --- | --- | --- |
| *Actin* | XM_024697950.1 | actin-F | GCCCCAATCAACCCAAAGT | **242** | **3.320** | **100.1** | **0.999** |
|  |  | actin-R | TAATCAGTCAAATCACGACCAGC |  |  |  |  |
| *bcacoaat1* | XM_024693202.1 | acoaat1-F | GCCTACAACAAGAAGGAGCACAT | **174** | **3.289** | **101.4** | **0.998** |
|  |  | acoaat1-R | GCCACCGCTAACTTCCACTG |  |  |  |  |
| *bcacoaat2* | XM_001559061.2 | acoaat2-F | AGCAGTCTTCCCCCATTCG | **156** | **3.444** | **95.1** | **0.999** |
|  |  | acoaat2-R | GATAGCGTATTCGTCTTGTTGTTCT |  |  |  |  |
| *bchmgs1* | XM_001552372.2 | hmgs1-F | CCAACCCAACCGCATCAG | **212** | **3.355** | **98.6** | **0.997** |
|  |  | hmgs1-R | CAACAAACCAACCAATCCACC |  |  |  |  |
| *bchmgs2* | XM_001557521.2 | hmgs2-F | CAAAGATAAGACGCTGGAGAAGAC | **212** | **3.413** | **96.3** | **0.993** |
|  |  | hmgs2-R | TAGCAATGCCACTTCCAAAACT |  |  |  |  |
| *bchmgr* | XM_001559909.2 | hmgr-F | GGTCAAGGGTAGCAGCATCC | **115** | **3.301** | **100.8** | **0.996** |
|  |  | hmgr-R | TTGAATACTGCTGTTGCCGC |  |  |  |  |
| *bcerg12* | XM_024692977.1 | erg12-F | GCATACACGGAAACCCATCTG | **185** | **3.472** | **94.1** | **0.991** |
|  |  | erg12-R | CAAATCCACTTTAGCAACCTCG |  |  |  |  |
| *bcerg8* | XM_001553881.2 | erg8-F | GGTCTTCTTAGCAGGAGGGTATCT | **209** | **3.24** | **103.5** | **0.997** |
|  |  | erg8-R | CTTCAATACCCCCATCATCCTTA |  |  |  |  |
| *bcmvd1* | XM_001546637.2 | mvd1-F | TCGCTTCTCCTCAATGGCTC | **189** | **3.275** | **102** | **0.996** |
|  |  | mvd1-R | AGCGGATGAAGCGAGACCT |  |  |  |  |
| *bcidi* | XM_024696269.1 | idi1-F | AATGACGGAAAATGGGGAGAG | **122** | **3.37** | **98** | **0.993** |
|  |  | idi1-R | TTGAGTCCGTCGGCAGAAA |  |  |  |  |
| *bcerg20* | XM_001558226.2 | erg20-F | TGTTCCCGACTCAGTATCTCTCC | **211** | **3.263** | **102.5** | **0.998** |
|  |  | erg20-R | GAGGAAAGCGTCGTTGATAGC |  |  |  |  |
| *bcaba1* | XM_024694601.1 | aba1-F | GTTCCTCTTTTCTCCCCCCTT | **193** | **3.269** | **102.3** | **0.997** |
|  |  | aba1-R | GCCTTCAACTTCGCACTCCT |  |  |  |  |
| *bcaba2* | XM_024694600.1 | aba2-F | GGCAAAGAACTCGTTGTCGG | **219** | **3.277** | **101.9** | **0.996** |
|  |  | aba2-R | GCGTGGAGGTAAGGCAAATC |  |  |  |  |
| *bcaba3* | XM_024694602.1 | aba3-F | GCCAGATTCACGATTGCCTC | **204** | **3.273** | **102.1** | **0.999** |
|  |  | aba3-R | ACACTCGCTGTAGGCTTTGATG |  |  |  |  |
| *bcaba4* | [XM_001553919.2](https://www.ncbi.nlm.nih.gov/nucleotide/XM_001553919.2?report=genbank&log$=nucltop&blast_rank=7&RID=J7HSDK7701R) | aba4-F | AGGAAGTTTGGAGAGGTGGAAG | **222** | **3.151** | **107.4** | **0.997** |
|  |  | aba4-R | CGAGCCGTTGTTAGCCATTAC |  |  |  |  |

**Table S3.** Concentration of antibiotic application

| Antibiotic | Working concentration (μg/mL) | | | | |
| --- | --- | --- | --- | --- | --- |
| G418, Geneticin | 20 | 40 | 80 | 160 | 320 |
| Bleomycin | 20 | 40 | 80 | 160 | 320 |
| Nourseothricin | 20 | 40 | 80 | 160 | 320 |
| Benomyl | 0.05 | 0.1 | 0.2 | 0.4 |  |
| The above antibiotics were prepared in sterile water. | | | | | |

**Table S4.** Endogenous overexpression of genes

| Gene symbol | Annotation | Length (bp) |
| --- | --- | --- |
| *HMGR* | 3-hydroxy-3-methylglutaryl-coenzyme A reductase(hypothetical protein) | 3642 |
| *MK* | mevalonate kinases | 1497 |
| *FPPS* | farnesyl pyrophosphate synthetase | 1044 |
| *ABA1* | Cytochrome P450 | 1530 |
| *ABA2* | Cytochrome P450 | 1584 |
| *ABA3* | Cyclization (PROVISIONAL) | 1323 |

# SUPPLEMENTARY FIGURES


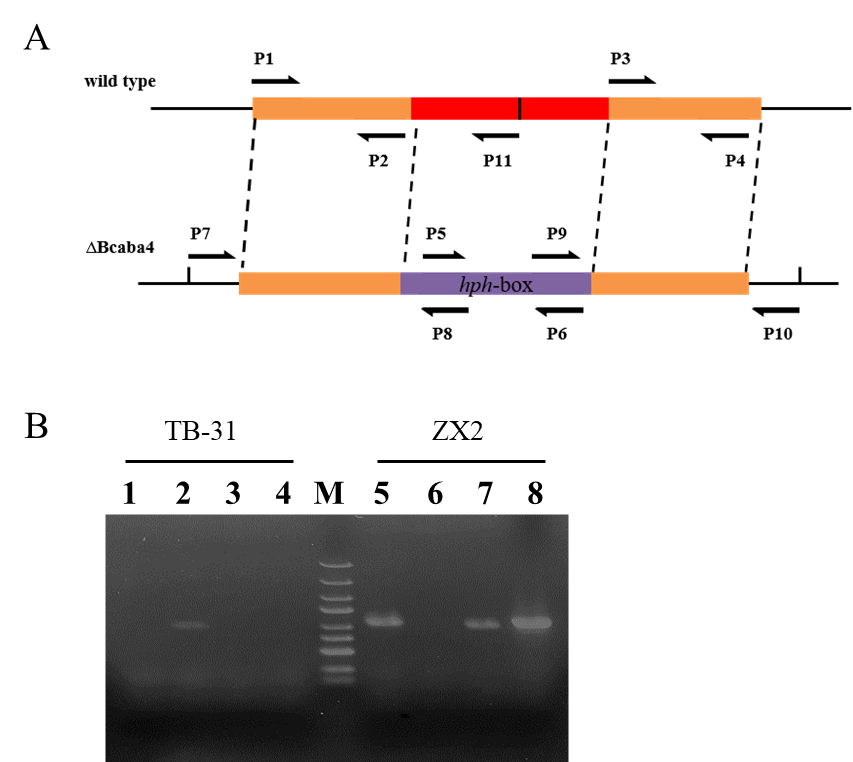


**Figure S1.** Identification of the mutant ZX2. (A) Schematic representation of the *bcaba4* deletion strategy. *bcaba4* and Hygromycin phosphotransferase fragment (*hph*) resistance box are denoted by red and purple. Black arrows indicate the gene-specific primers. (B) Identification of *bcaba4* gene knockout in ZX2 strain. M: 5000 bp DNA Marker, Lane1/5: p7/p8 amplified the upstream of *bcaba4* and promoter of replacement cassette, Lane2/6: p12/p13 amplified *bcaba4* ORF, Lane3/7: p9/p10 amplified the downstream of *bcaba4* and terminator of replacement cassette, Lane4/8: p5/p6 amplified *hph*.


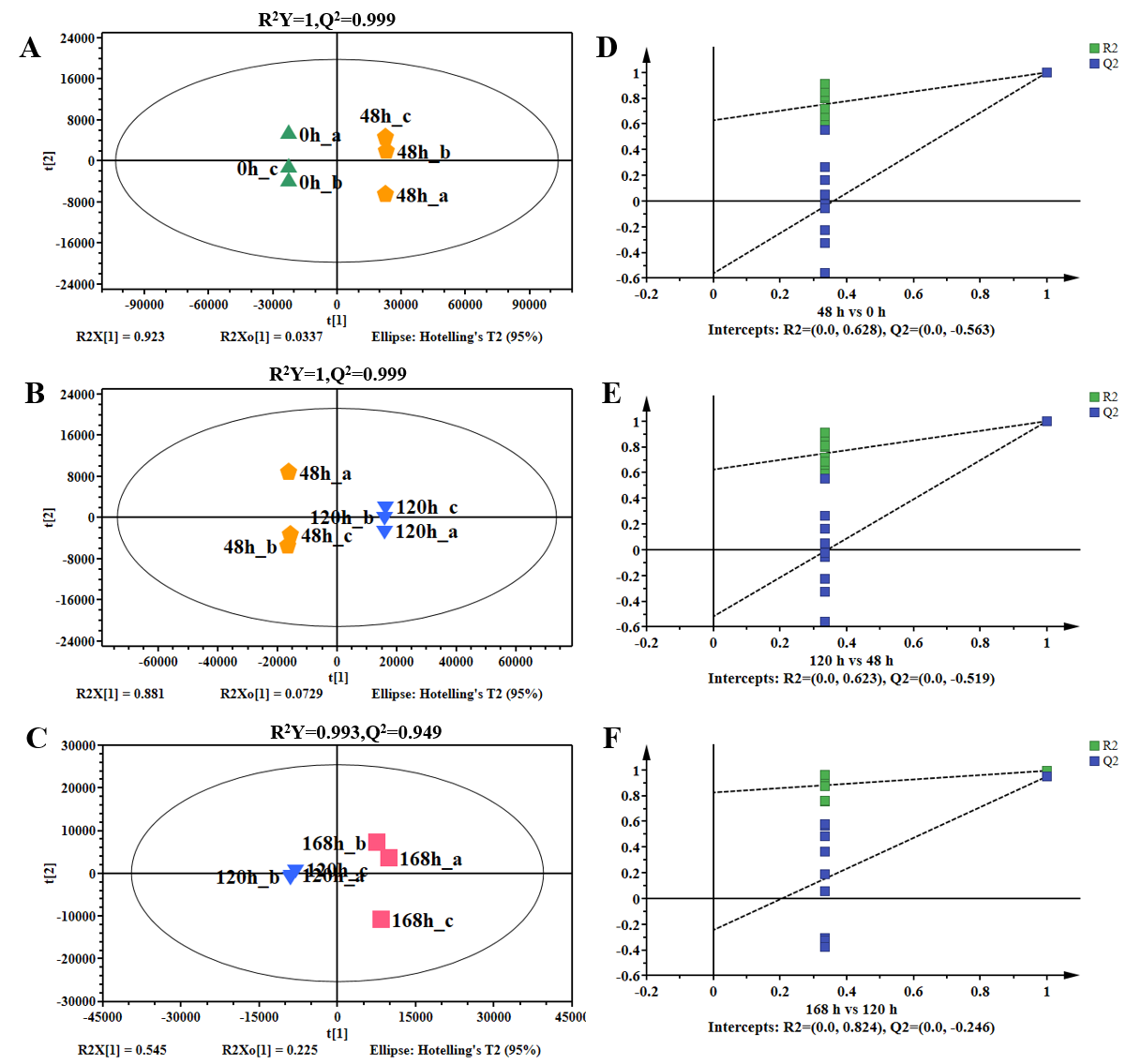


**Figure S2.** OPLS-DA score chart and permutation test (200 times).


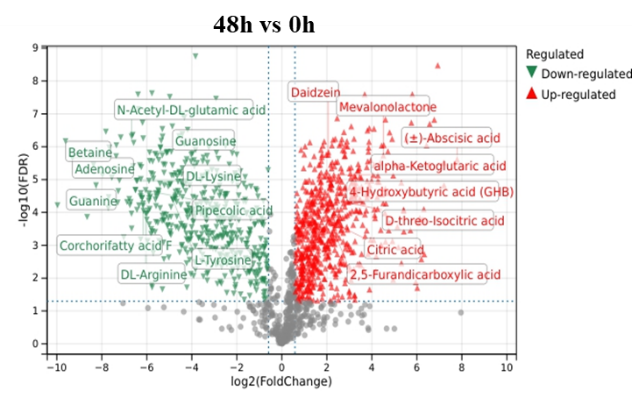

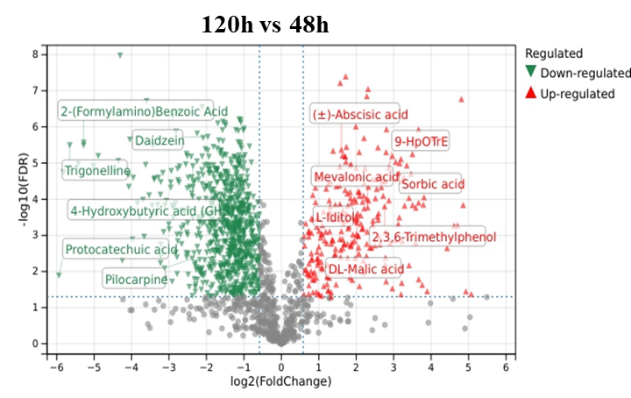


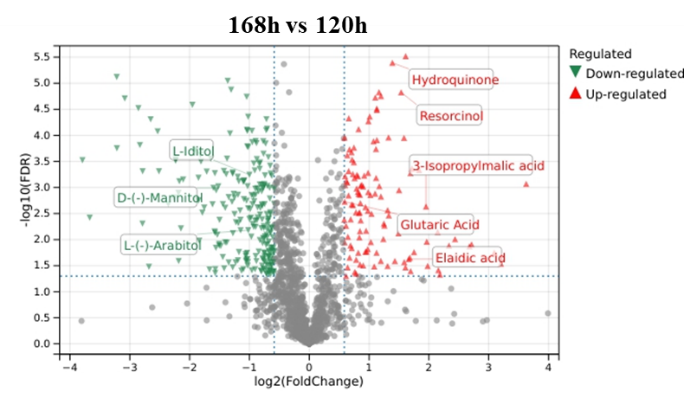


**Figure S3.** Volcano plot of differential metabolites between different fermentation stages


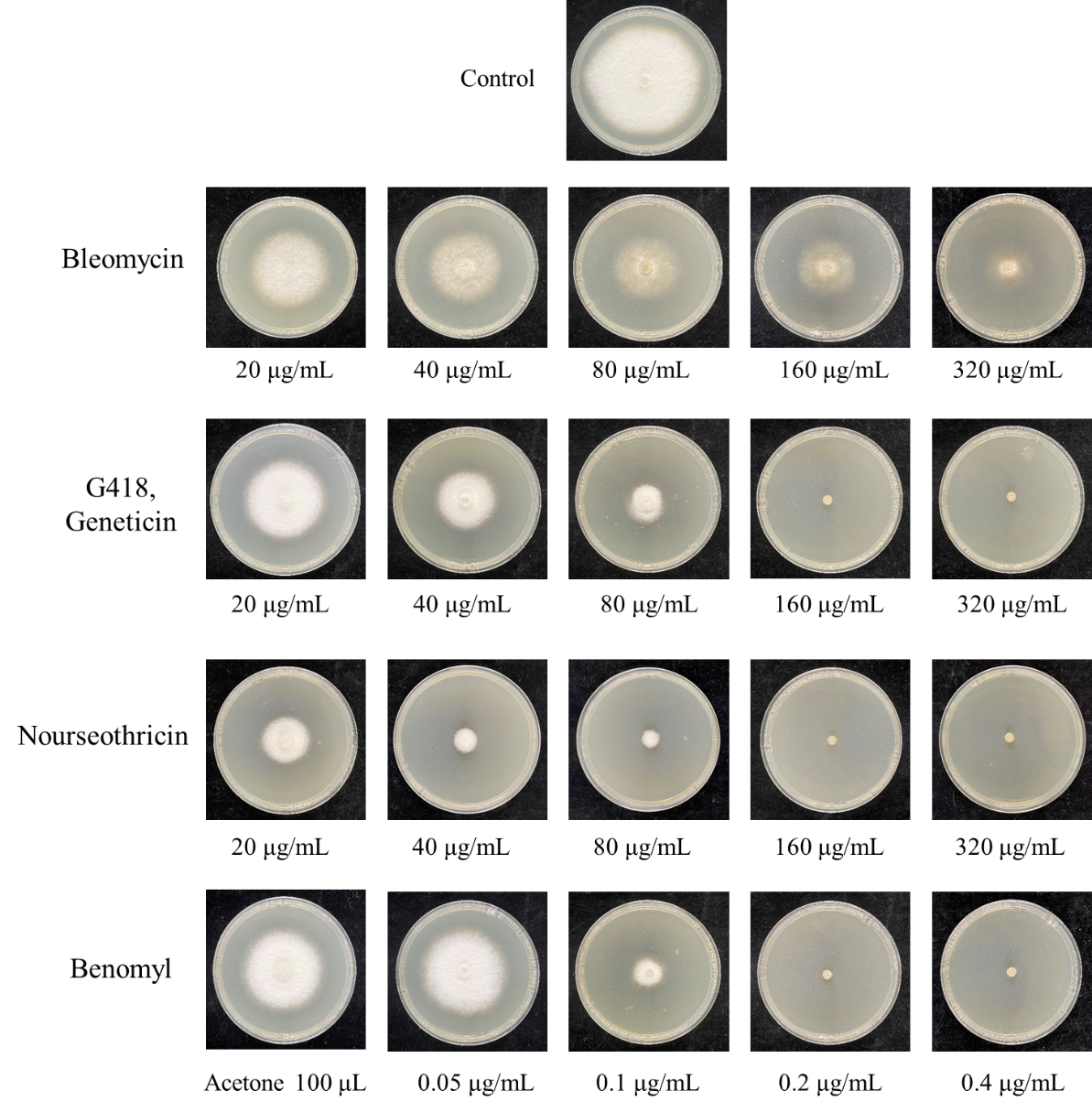


**Figure S3.** *B.cinerea* ZX2 antibiotic sensitivity test. All strain were inoculated on potato dextrose agar (PDA) plates in the presence of antibiotic and cultured at 25 ℃ for 5 days.


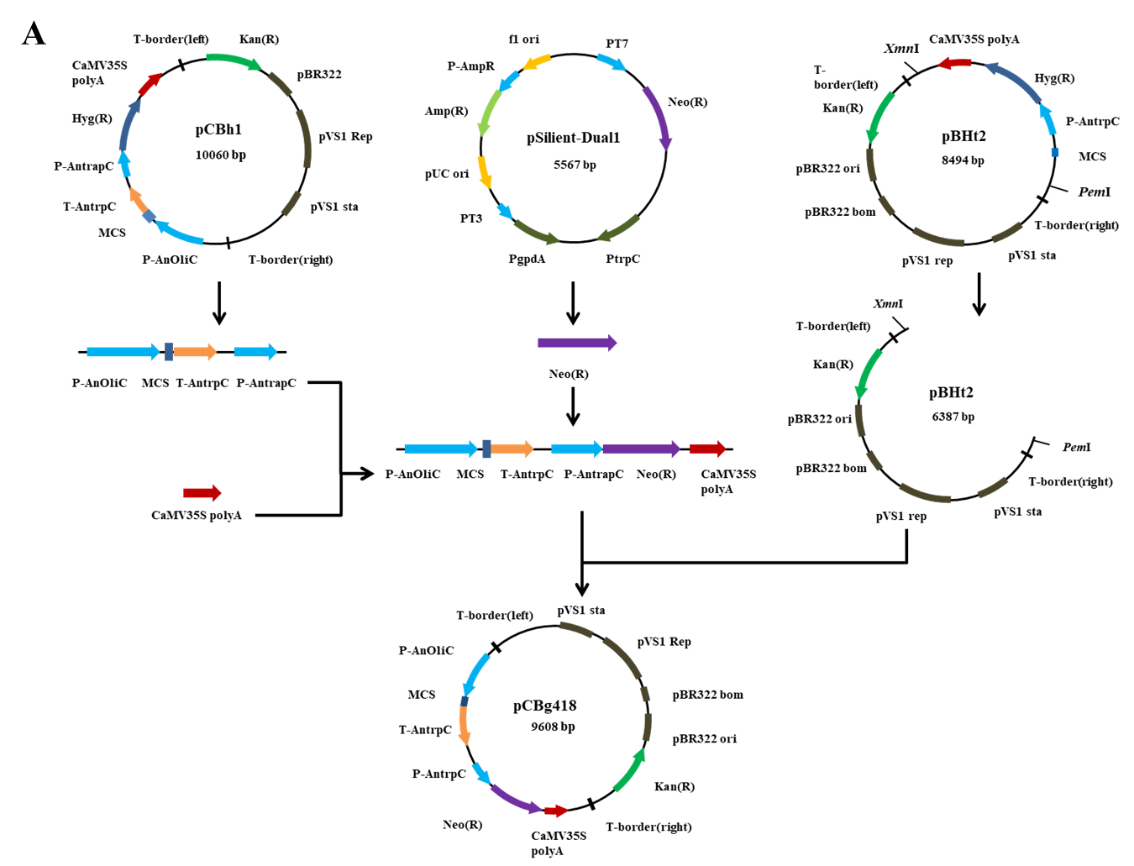


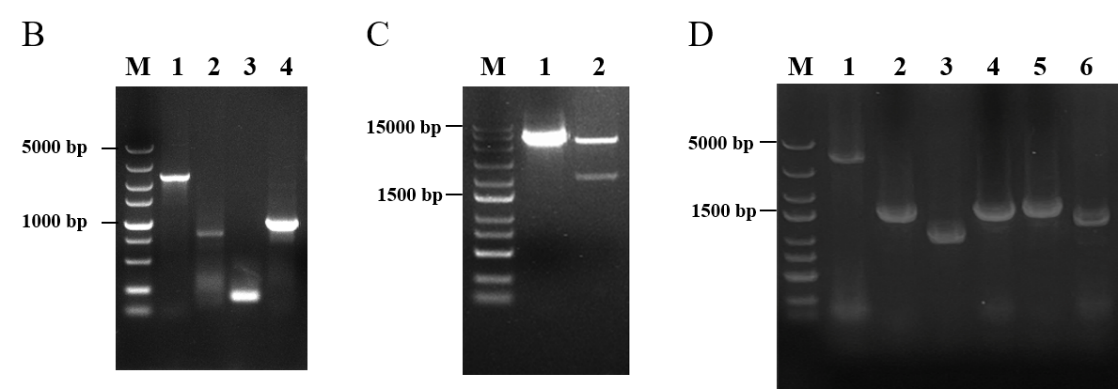


**Figure S4.** Construction of overexpression vector pCBg418 and overexpression vector with target genes. (A) Schematic build process of the over-expression vector pCBG418. (B) Overexpression vector components. M: 5000 bp DNA Marker, Lane 1-4: MCS expression cassette+AnTrpC promoter (2250 bp), G418 resistance gene (795 bp), CaMV35S polyA signal (211bp), G418 resistance gene+CaMV35S polyA (1006 bp).(C) Overexpression vector backbone. M: 15000 bp DNA Marker, Lane 1-2: pBht2; pBht2 (*Pme*I and *Xmn*I digests). (D) Overexpressed gene fragment amplified by cDNA. M: 5000 bp DNA Marker, Lane 1-6: *bchmgr*, *bcerg12*, *bcrrg20*, *bcaba1*, *bcaba2* and *bcaba3*.

The template used for amplifying the cloning site (MCS) cassette and the Aspergillus nidulans trpC promoter was pCBh1, with primer pairs pCBh1-AnoliC and pCBh1-AntrpC. The reverse primer pCBh1-AntrpC used here contains an additional 18 bp oligonucleotide homologous to the resistance gene neo in pSilient-Dual1. The coding domain sequence (CDS) of the resistance gene neo was used as a template for pSilent-Dual, with the forward primer neo-F containing an additional 18 bp oligonucleotide identical to the 18 bp DNA sequence at the 3' end of AntrpC in pBCh1. The reverse primer neo-R also contains an 18 bp oligonucleotide corresponding to the DNA sequence at the 5' end of CaMV35S-polyA in plasmid pCBh1. CaMV35S-polyA was amplified from pCBh1 using primer pair CaMV35S-F/CaMV35S-R. The reverse primer CaMV35S-R contains the same 18bp DNA sequence as that following the XmnI site of pBHt2 (excluding XmnI). The three PCR products described above were ligated by bridging PCR to produce a PCR product that included two expression cassettes: the G418 resistance cassette and the MCS cassette (Fig. S4).


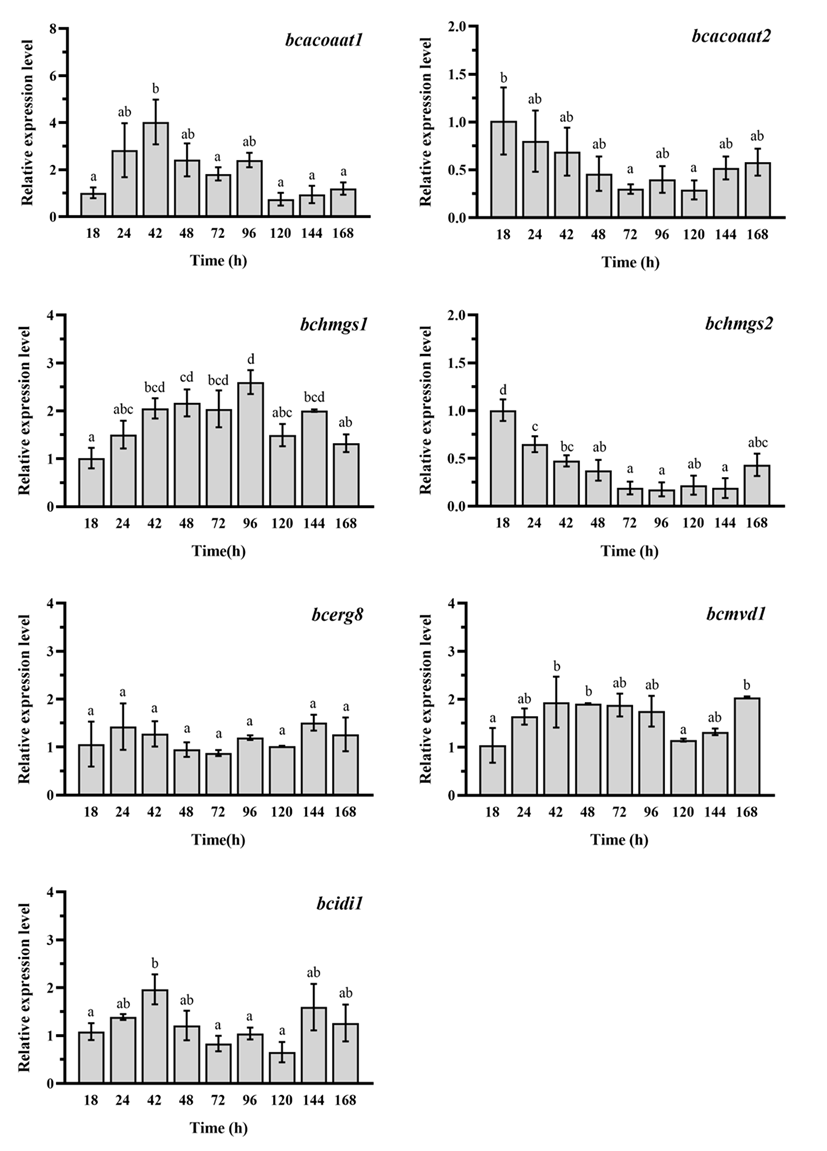


**Figure S5.** Expression of genes related to 1´,4´-*trans*-ABA-diol synthesis during 50 L fed-batch fermentation. *acoaat1*:acetyl-CoA acetyltransferase 1; ACOAAT2: acetyl-CoA acetyltransferase 2; HMGS, hydroxymethylglutaryl-CoA synthase; HMGR: geranylgeranyl pyrophosphate synthase; MK: mevalonate kinase; PMK: phosphomevalonate kinase; PMVADO: diphosphomevalonate decarboxylase; IDI: isopentenyl-diphosphate delta-isomerase; FPPS: farnesyl diphosphate synthase. BcABA1: *B. cinerea* cytochrome P450; BcABA2, *B. cinerea* cytochrome P450; BcABA3: *B. cinerea* α-ionylideneethane synthases.

**
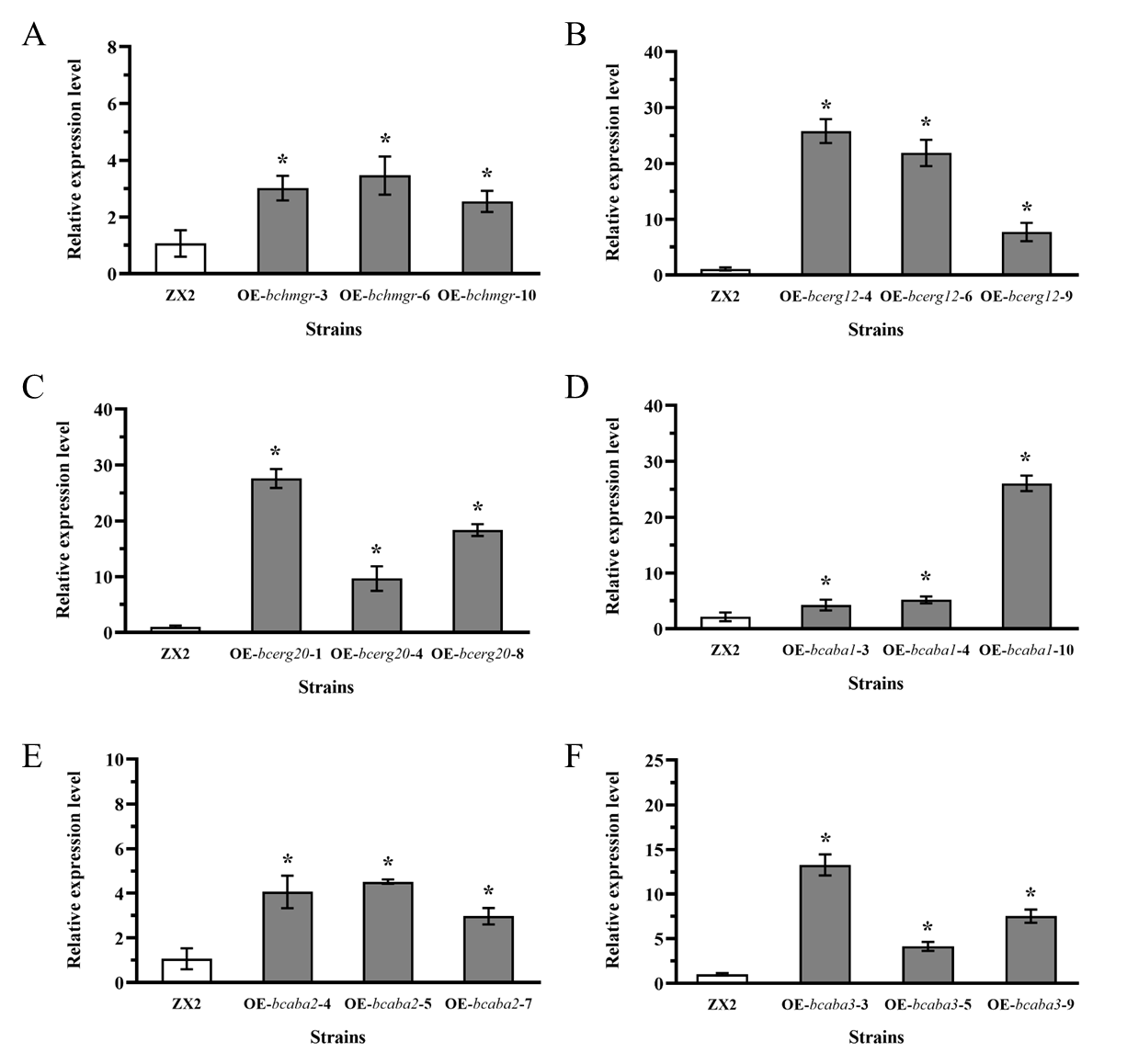
**

**Figure S6.** RT-qPCR validation of the relative gene expression of single gene over-expression strains. (A) ZX2 HR strain; (B) ZX2 MK strain; (C) ZX2 FS strain; (D) ZX2 A1 strain; (E) ZX2 A2 strain; (F) ZX2 A3 strain. Three single-gene overexpression mutants were randomly selected from each group and cultured on PDA media at 25°C for 7 days, and RNA was extracted for qRT-PCR analysis.
